# Function conservation and disparities of zebrafish and human LGP2 genes in fish and mammalian cells responsive to poly(I:C)

**DOI:** 10.3389/fimmu.2022.985792

**Published:** 2022-08-17

**Authors:** Xiu-Ying Gong, Zi-Ling Qu, Yi-Lin Li, Hao-Yu Sun, Xiang Zhao, Cheng Dan, Jian-Fang Gui, Yi-Bing Zhang

**Affiliations:** ^1^State Key Laboratory of Freshwater Ecology and Biotechnology, Institute of Hydrobiology, Chinese Academy of Sciences, Wuhan, China; ^2^College of Advanced Agricultural Science, University of Chinese Academy of Sciences, Beijing, China; ^3^The Innovation Academy of Seed Design, Chinese Academy of Sciences, Wuhan, China

**Keywords:** LGP2, IFN response, dual regulation, function switch, poly(I:C)

## Abstract

Retinoic acid inducible gene-I (RIG-I)-like receptors (RLRs) are viral RNA sensors that regulate host interferon (IFN)-mediated antiviral signaling. LGP2 (laboratory genetics and physiology 2) lacks the N-terminal caspase activation and recruitment domains (CARDs) responsible for signaling transduction in the other two RLR proteins, RIG-I and melanoma differentiation associated gene-5 (MDA5). How LGP2 regulates IFN signaling is controversial, and inconsistent results have often been obtained in overexpression assays when performed in fish cells and mammalian cells. Here we report that the differential sensitivity of fish cells and mammalian cells to poly(I:C) transfection conceals the function conservation of zebrafish and human LGP2. In fish cells, overexpression of zebrafish or human LGP2 initially activates IFN signaling in a dose-dependent manner, followed by inhibition at a critical threshold of LGP2 expression. A similar trend exists for LGP2-dependent IFN induction in response to stimulation by low and high concentrations of poly(I:C). In contrast, overexpression of zebrafish or human LGP2 alone in mammalian cells does not activate IFN signaling, but co-stimulation with very low or very high concentrations of poly(I:C) shows LGP2-dependent enhancement or inhibition of IFN signaling, respectively. Titration assays show that LGP2 promotes MDA5 signaling in mammalian cells mainly under low concentration of poly(I:C) and inhibits RIG-I/MDA5 signaling mainly under high concentration of poly(I:C). Our results suggest that fish and human LGP2s switch regulatory roles from a positive one to a negative one in increasing concentrations of poly(I:C)-triggered IFN response.

## Introduction

Laboratory of genetics and physiology 2 (LGP2, or DHX58), retinoic acid inducible gene-I (RIG-I, or DDX58) and melanoma differentiation associated gene-5 (MDA5, or IFIH1) constitute a group of RIG-I-like receptors (RLRs) regulating interferon (IFN)-mediated antiviral signaling (1). All three RLR proteins share a homologous DExD/H-box RNA helicase domain in the central regions and a regulatory domain (CTD) in the C-termini; however, LGP2 lacks the two N-terminal caspase activation and recruitment domains (CARDs) responsible for signaling transduction in both RIG-I and MDA5 (2). Cytosolic viral-derived RNA recognition by RIG-I and MDA5 is accompanied by CARD phosphorylation, enabling their interaction with the CARDs of the adaptor mitochondrial antiviral signaling protein (MAVS, also known as IPS1, VISA or CARDIF) to initiate a signal transduction, which finally activates IFN regulatory factor 3/7 (IRF3/7) for the expression of IFN and downstream IFN-stimulated genes (ISGs) (1).

In mammals, LGP2 is initially identified as a feedback inhibitor of RIG-I/MDA5-trigged IFN signaling, based on its stronger RNA binding affinity than MDA5 or RIG-I and its virus-inducible features (3–5); however, further evidences indicate that both RNA binding ability and ATP hydrolysis activity do not contribute to the negative regulation of LGP2 (6, 7). Surprisingly, the negative role of LGP2 is supported by the initial strain of LGP2^-/-^ mice (8), but refuted by the subsequent strain of LGP2^-/-^ mice, the latter of which claims a positive role of LGP2 in RLR signaling (9). Delineation of a third knockout strain reveals an essential role of LGP2 in controlling CD8^+^ T cell survival and fitness in response to viral infection but not in innate antiviral response (10). Additionally, LGP2-transgenic mice exhibit increased survival advantages but decreased IFN response upon viral infection (11, 12).

Despite that these *in vivo* studies largely confuse the understanding of LGP2 function, the former two strains of LGP2^-/-^ mice are more susceptible to encephalomyocarditis virus (EMCV) infection (sensed by MDA5) (8, 9), supporting a synergistic role of LGP2 in MDA5-mediated IFN signaling. This notion is further evidenced by the finding that MDA5 recognition of EMCV RNA depends on LGP2 with intact RNA biding activity and ATP hydrolysis activity (13). Consistently, LGP2 enhances the interaction between MDA5 and dsRNA (14–16), and thus promotes exposure of MDA5’s CARDs for MAVS signaling, through facilitating MDA5 fiber assembly by incorporation into the fibers and inducing significant conformational changes on MDA5 (17). Titration of LGP2 expression suggests a dose-dependent model (2): low levels of LGP2 synergize with MDA5 as a positive regulator, but high levels of LGP2 act as an inhibitor of RIG-I and MDA5 signaling (7, 15, 16). Further mechanism studies show that LGP2 downregulates IFN response by blocking the interaction between RIG-I and MAVS (18), or inhibiting Dicer-mediated processing of dsRNA (19), or interfering with the function of TRAF ubiquitin ligases (20). Additionally, LGP2 is believed to interact with PACT, a cofactor of DICER in the processing and biogenesis of microRNAs, respectively regulating RIG-I- and MDA5-mediated IFN signaling (21). However, these studies cannot perfectly interpret how LGP2 exerts antithetic effects toward virus infection.

Fish has all three RLR receptors and downstream signal molecules, including MAVS, TBK1, IRF3, IRF7 (22–25). In mammals, overexpression of RIG-I stimulates a weak activation of IFN signaling but a strong one in the presence of co-stimulation by virus infection or poly(I:C) (polyinosinic-polycytidylic acid) transfection (3–5, 26, 27). However, overexpression of fish RIG-I, MDA5 or LGP2 alone in fish cells directly gives efficient regulator activity (24, 28, 29), although there is an exception that overexpression of flounder LGP2 stimulates IFN response with the requirement of virus infection (30). Similarly, the earlier documents showed opposing function of fish LGP2 (24, 28, 30–35). Using overexpression strategies, we subsequently found that overexpression of zebrafish LGP2 alone stimulates fish IFN response and particularly, it shifts regulatory roles from an initially positive one to a following negative one in fish cells during virus infection or poly(I:C) transfection (29). Recently we have provided *in vivo* evidence for function switch of zebrafish LGP2 toward virus infection (36).

Given the conservation of fish and mammalian LGP2 proteins, we wonder whether the function switch of LGP2 happens in mammalian cells under the same experimental conditions. In the present study, we compared LGP2-mediated regulation of IFN response in fish cells and mammalian cells in the absence or presence of poly(I:C). We found that fish cells and mammalian cells were differentially sensitive to poly(I:C) transfection, which might contribute to the different results in both cells when the same assays were performed. In fish cells, human LGP2 (HsLGP2) acted like zebrafish LGP2 (DrLGP2), stimulating IFN response when overexpressed alone or in the presence of low concentrations of poly(I:C) but inhibiting IFN response triggered by higher concentrations of poly(I:C). In mammalian cells, DrLGP2 functioned as HsLGP2, triggering IFN response with supplementary stimulation by poly(I:C) at low concentrations, particularly in the presence of MDA5; however, they displayed inhibitory regulation of IFN response triggered by RIG-I or MDA5 in the presence of poly(I:C) at high concentrations. Our results suggest that fish and human LGP2s indeed harbor similar abilities to play dual regulation of IFN response in fish cells and mammalian cells although they are differentially sensitive to dsRNA.

## Materials and methods

### Cells and poly(I:C)

Two fish cells, epithelioma papulosum cyprini cells (EPC) were from ATCC (CRL-2872), and grass carp *Ctenopharyngodon idella* ovary cells (CO) were established in 1978 by Institute of Hydrobiology, Chinese Academy of Sciences. Two mammalian cells were derived from ATCC (HEK293T: CRL-3216; COS7: CRL-1651). Fish cells were grown at 28°C in medium 199 supplemented with 10% fetal bovine serum (FBS), and mammalian cells cultured in DMEM basic (Gibco) with 10% FBS at 37°C in a humidified incubator containing 5% CO_2_. The medium molecular weight (MMW) poly(I:C) was purchased from SIGMA (Catalog no. I3036), and the high molecular weight (HMW) poly(I:C) from Enzo Life Sciences (Catalog no. ALX-746-021). Until indicated, the poly(I:C) used in the study was the MMW poly(I:C). All experiments were approved by the Animal Care and Use Committee of Institute of Hydrobiology, Chinese Academy of Sciences.

### Plasmids

Tag-free expression plasmids of zebrafish LGP2 (DrLGP2) (29) and human LGP2 (HsLGP2) were made by insertion of corresponding ORFs into pcDNA3.1(+) vector (Invitrogen). The ORF of human LGP2 is amplified from an expression plasmid provided by Professor Jin Zhong from Institute Pasteur of Shanghai, Chinese Academy of Sciences (37). Similarly, Tag-free plasmids of human MDA5 (HsMDA5) and RIG-I (HsRIG-I) were generated by inserting their ORFs into EcoRV site of pcDNA3.1(+) vector (Invitrogen). HA-tagged plasmids (DrLGP2-HA, HsLGP2-HA, HsIRF3-HA) were generated by inserting the ORFs into EcoRV site of pcDNA3.1(+) vector (Invitrogen) that had preexisted a HA coding sequence into NotI site. DrMDA5, DrRIG-I, DrIRF3-HA were described previously (38, 39). Crucian carp IFNpro-luc (CaIFNpro-luc) and zebrafish IFNφ1pro-luc (DrIFNφ1pro-luc) were reported previously (23, 24). Human IFNβpro-luc (HsIFNβpro-luc) was kindly provided by Professor Hongbin Shu from Wuhan University (40).

### Luciferase activity assays

Transfection assays were performed with polyethylenimine, linear (PEI, MW25000; Aldrich, 1μg/μl of storage concentration) according to our studies previously (36, 41, 42). Typically, fish cells were seeded overnight in 24-wells plates, transfected with various plasmids at a ratio of 10:10:1 (promoter-driven luciferase plasmid/expression plasmid/Renilla luciferase plasmid pRL-TK). In mammalian cells, the ratio of transfected plasmids is changed to 1000:1000:1 due to relative low luciferase activity when the same doses were used. If necessary, cells were transfected again with poly(I:C) at 24 h post the first round of transfection. Total amounts of plasmid DNAs were kept constant in all wells by supplementing with empty vectors. Until noted, the transfected plasmids were tag-free. Luciferase activities were measured by a Junior LB9509 luminometer (Berthold, Pforzheim, Germany), according to the Dual-Luciferase Reporter Assay System (Promega, USA). All results were shown as a representative of more than three independent experiments, each performed in triplicate.

### RNA extraction, cDNA synthesis, and quantitative real-time PCR

Cells seeded overnight in 12-wells plates were transfected with various constructs with 2-fold doses over those in 24-wells plates. Total RNA was extracted by TRIZOL Reagent (TIANMO BIOTECH, China), followed by treatment with RNase-free DNase I to remove genomic DNA. First-strand cDNA was synthesized using random primers or Oligo(dT)_20_VN (Monad, China). RT-qPCR was performed with Universal Blue qPCR SYBR Green Master Mmix (YEASEN, China) in a DNA Engine Chromo 4 real-time system (BioRad, USA). All samples were analyzed in triplicate and the expression value of target genes was normalized to β-actin (43). All results were shown as a representative of more than three independent experiments, each performed in triplicate. The primers used in this study were listed in **Supplemental Table 1**.

### DNA/RNA pulldown assays

DNA pulldown assays were performed as described previously (38, 39). Cells were seeded overnight in 10 cm dishes and transfected with various expression constructs. 24 h later, cells were collected in HKMG buffer with protease inhibitor (Roche) and lysed by ultrasound treatment. 10% of cell lysates were taken as input. Appropriate cell lysates were incubated for 24h with promoter DNA probe-bound beads, which were pre-made by mixing 30 μl M-280 streptavidin Dynabeads (Invitrogen) and 100 ng of biotinylated DNA in 1×BW buffer for 30 min. The beads were washed 5 times with HKMG buffer, added with 1×SDS loading buffer and boiled for 10 min, followed by western blotting. RNA pulldown was similar to DNA pulldown, except for all reagents in DEPC water containing RNA enzyme inhibitor (Thermo Fisher Scientific).

### Nuclear and cytoplasmic protein extraction

Nuclear and cytoplasmic proteins were extracted according to the manufacturer’s protocol (YEASEN, China) and our previous paper (24, 36). Briefly, cells were added with PBS buffer and collected by cell scrapers. After centrifugation, cell pellets were treated with reagent A containing PMSF, vortexed thoroughly, incubated on ice for 10-15 min, finally added with cytoplasmic protein extraction reagent B. After another round of vortex and centrifugation, supernatants were collected as cytoplasmic proteins. The remaining pellets were further incubated with reagent C containing PMSF for 30 min on ice, vortexed thoroughly, and centrifuged to obtain nuclear proteins.

### Coimmunoprecipitation and Western blotting

Co-immunoprecipitation (Co-IP) assays and western blotting were performed as previously described (23, 29, 36, 44). Antibodies specific to Lamin A/C, α-tubulin and β-actin were purchased from Cell Signaling Technology (USA). Antibodies of crucian carp IRF3 (23) was described previously. Zebrafish LGP2-specific Ab was generated by immunization of rabbits with a purified peptide corresponding to 192-417 aa of zebrafish LGP2 (36).

### Statistical analysis

Statistical analysis was conducted with Student’s t-test and ANOVA for the data derived from luciferase assays and RT-PCR assays.

## Results

### Overexpression of zebrafish or human LGP2 alone directly activates IFN response in fish cells but not in mammalian cells

**Figure 1:**
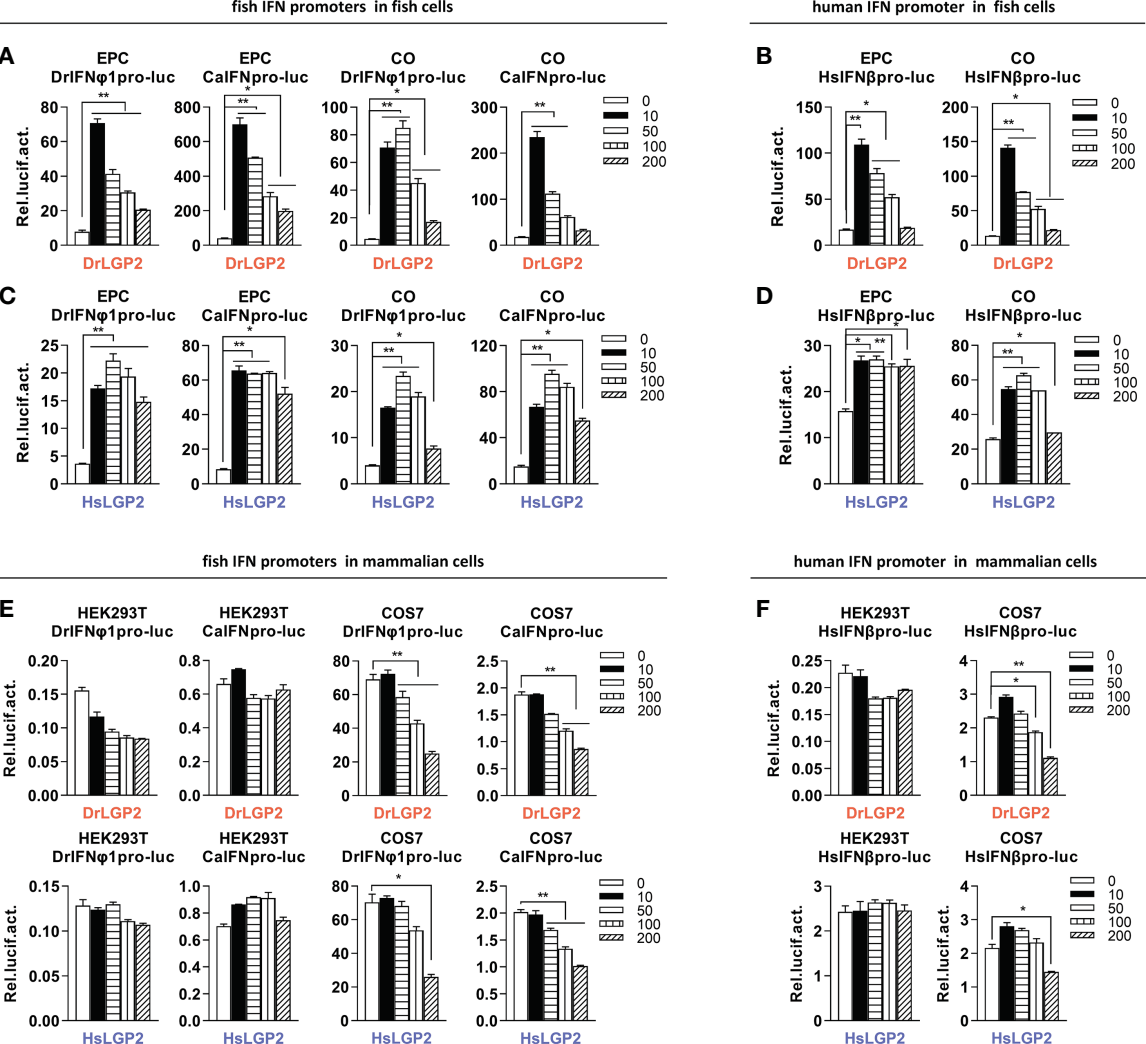
Overexpression of zebrafish or human LGP2 facilitates the activation of fish and human promoters in fish cells but not in mammalian cells **(A–D)** DrLGP2 and HsLGP2 both activated fish IFN promoters **(A, C)** and human IFNβ promoter **(B, D)** in fish cells. EPC cells and CO cells seeded overnight in 24-wells plates were co-transfected with DrIFNφ1pro-luc or CaIFNpro-luc **(A, C)**, or HsIFNβpro-luc (200 ng) **(B, D)**, together with DrLGP2 **(A, B)** or HsLGP2 **(C, D)** at increasing doses (0, 10, 50, 100, 200 ng). pRL-TK (20 ng) was transfected as internal control. 48 h later, cells were collected for luciferase assays. **(E, F)** DrLGP2 and HsLGP2 did not activate fish IFN promoters **(E)** and human IFNβ promoter **(F)** in mammalian cells. HEK293T cells and COS7 cells seeded overnight in 24-wells plates were co-transfected with DrIFNφ1pro-luc or CaIFNpro-luc **(E)**, or HsIFNβpro-luc (200 ng) **(F)**, together with DrLGP2 or HsLGP2 at increasing doses (0, 10, 50, 100, 200 ng). Renilla vector (pRL-TK, 0.2 ng) was transfected as internal control. 48 h later, cells were collected for luciferase assays. Error bars show the SDs of triplicate transfections. Data were shown as mean ± SD (N=3). *P* values were calculated using ANOVA. **P < 0.01, *P < 0.05.

**Figure 2:**
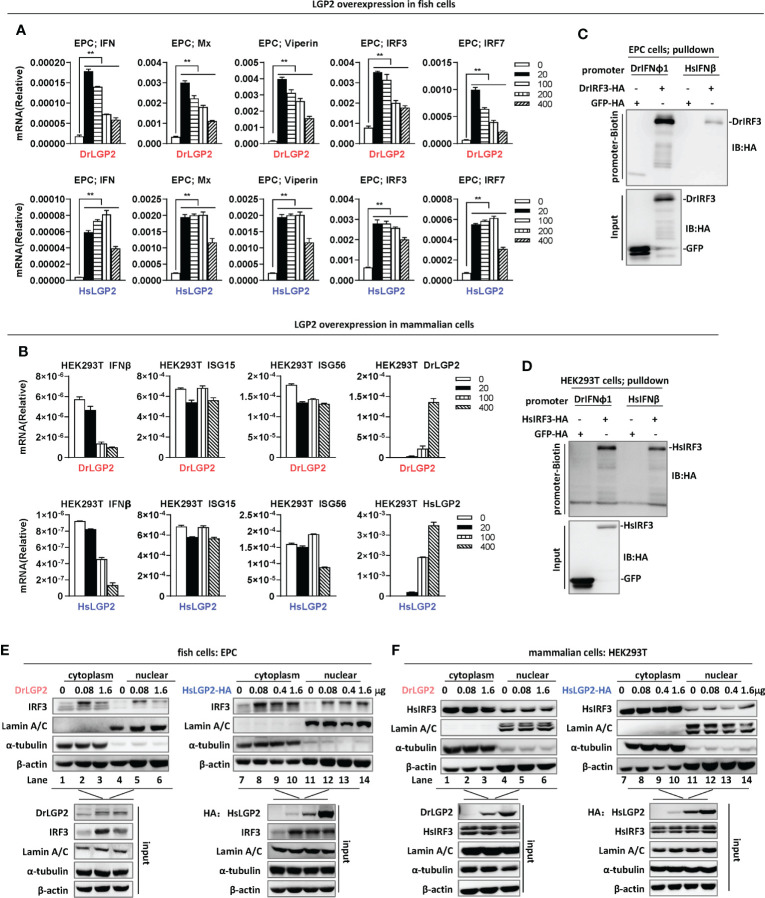
Overexpression of zebrafish or human LGP2 induces IFN response in fish cells but not in mammalian cells **(A, B)** RT-PCR analysis of transcriptional levels of IFN and ISGs induced by DrLGP2 and HsLGP2 in EPC cells **(A)** and HEK293T cells **(B)**. EPC cells **(A)** and HEK293T cells **(B)** seeded overnight in 12-wells plates were transfected with DrLGP2 or HsLGP2 at increasing doses (0, 20, 10, 200, 400 ng) for 24 h, followed by RT-PCR detection of cellular gene transcription. *P* values were calculated using ANOVA. **P < 0.01. **(C, D)** DNA pull-down assays verified the binding of DrIFNφ1 and HsIFNβ promoter DNA to DrIRF3 in EPC cells **(C)** and HsIRF3 in HEK293T cells **(D)**. EPC cells **(C)** and HEK293T cells **(D)** seeded in 10 cm dishes were transfected with DrIRF3-HA **(C)** or HsIRF3-HA **(D)**. GFP-HA was transfected in parallel as control. 24 h later, cells were lysed. One-tenth of cell lysates were taken as input, and the remaining was incubated overnight with 100 ng biotinylated DrIFNφ1 promoter DNA (-596 to +38) **(C)** or HsIFNβ promoter DNA (-338 to +93) **(D)**. The DNA-bound protein complexes were detected by western blots with anti-HA antibody. **(E, F)** overexpression of LGP2 increased nuclear IRF3 protein levels in fish cells but not in mammalian cells. EPC cells **(E)** and HEK293T cells **(F)** seeded in 5 cm dishes were transfected with DrLGP2 or HsLGP2 at increasing doses. 24 h later, cells were collected for nuclear and cytoplasmic separation, followed by western blot analyses of the indicated proteins using corresponding antibodies. The expression of Lamin A/C and α-tubulin verified the successful separation of nuclear and cytoplasmic lysates.

Given that overexpression of DrLGP2 alone is able to activate IFN response (29), we compared the stimulatory potential of DrLGP2 and HsLGP2 to fish and human IFN promoters in fish cells (EPC and CO, derived from common carp and grass carp, respectively) and mammalian cells (HEK293T and COS7, derived from human and monkey, respectively). It showed that overexpression of DrLGP2 in fish cells directly activated two fish IFN promoters (DrIFNφ1pro-luc and CaIFNpro-luc), with a stimulator potential contrary to the doses of DrLGP2 (10, 50, 100, 200 ng) (**Figure 1A**). Similar induction trends were observed using human IFNβ promoter (HsIFNβpro-luc) (**Figure 1B**). Sequence comparison showed that zebrafish and human LGP2 proteins harbor the conserved domain arrangements and secondary structures (**Supplementary Figure 1**). Interestingly, overexpression of HsLGP2 in two fish cell lines also directly activated two fish IFN promoters (**Figure 1C**), and human IFNβ promoter (**Figure 1D**), showing a similar pattern to that induced by DrLGP2. On the contrary, there was almost no activation of DrIFNφ1pro-luc and CaIFNpro-luc in HEK293T cells by either DrLGP2 or HsLGP2 at increasing doses compared to the control (**Figure 1E**, left four panels); and in COS7 cells, the basal activity of fish IFN promoters was even diminished with increasing doses of LGP2 (**Figure 2E**, right four panels). Similar results were seen when both mammalian cell lines were transfected with HsIFNβpro-luc (**Figure 1F**).

Subsequent RT-PCR assays showed that overexpression of either DrLGP2 or HsLGP2 significantly upregulated the transcription of cellular *ifn* and ISGs (*mx, viperin, irf3, irf7*) in EPC cells, with a pattern similar to that by luciferase assays (**Figure 2A**). However, similar overexpression could not induce the expression of *ifnb, isg15* and *isg56* in HEK293T cells (**Figure 2B**). Given that fish LGP2 triggers IFN response through IRF3 activation (29), DNA pulldown assays showed that either zebrafish IFNφ1 promoter DNA (DrIFNφ1pro, -596 to +38) or human IFNβ promoter DNA (HsIFNβpro, -338 to +93) bound to zebrafish IRF3 (DrIRF3) in EPC cells (**Figure 2C**), and also to human IRF3 (HsIRF3) in HEK293T cells (**Figure 2D**).

To strengthen the findings above, nuclear-cytoplasmic separation experiments were performed to detect nuclear translocation of IRF3, a landmark event of RLR-triggered IFN response (23, 42). Successful nuclear-cytoplasmic separation was verified by detection of Lamin A/C only in nucleus, and α-tubulin mostly in cytoplasm (**Figure 2E**). Fish IRF3 is a typical IFN-inducible protein (23, 42). Consistently, overexpression of DrLGP2 alone in EPC cells increased the protein level of IRF3 in cytoplasm (lanes 2 and 3 versus lane 1 in **Figure 2E**) and also in nucleus (lanes 5 and 6 versus lane 4 in **Figure 2E**), indicating that DrLGP2 alone indeed activates IFN response in fish cells. Similarly, fish IRF3 protein was upregulated in EPC cells when transfected with HsLGP2 alone (lanes 8-10 versus lane 7 in cytoplasm, lanes 12-14 versus lane 11 in nucleus in **Figure 2E**). Human IRF3 is not induced by IFN and IFN stimuli (23). Consistent protein levels of human IRF3 were detected in either cytoplasm (lanes 1-3) or nucleus (lanes 4-6) of HEK293T cells when transfected with DrLGP2 at increasing doses (**Figure 2F**, left panel), showing no activation of IFN response when DrLGP2 was overexpressed. Similar results were replicated in HEK293T cells when HsLGP2 was overexpressed (**Figure 2F**, right panel). These results together indicate that overexpression of zebrafish and human LGP2s directly activates IFN response in fish cells but not in mammalian cells.

### Zebrafish and human LGP2s play antithetic roles in regulating IFN response by poly(I:C) in fish cells

**Figure 3:**
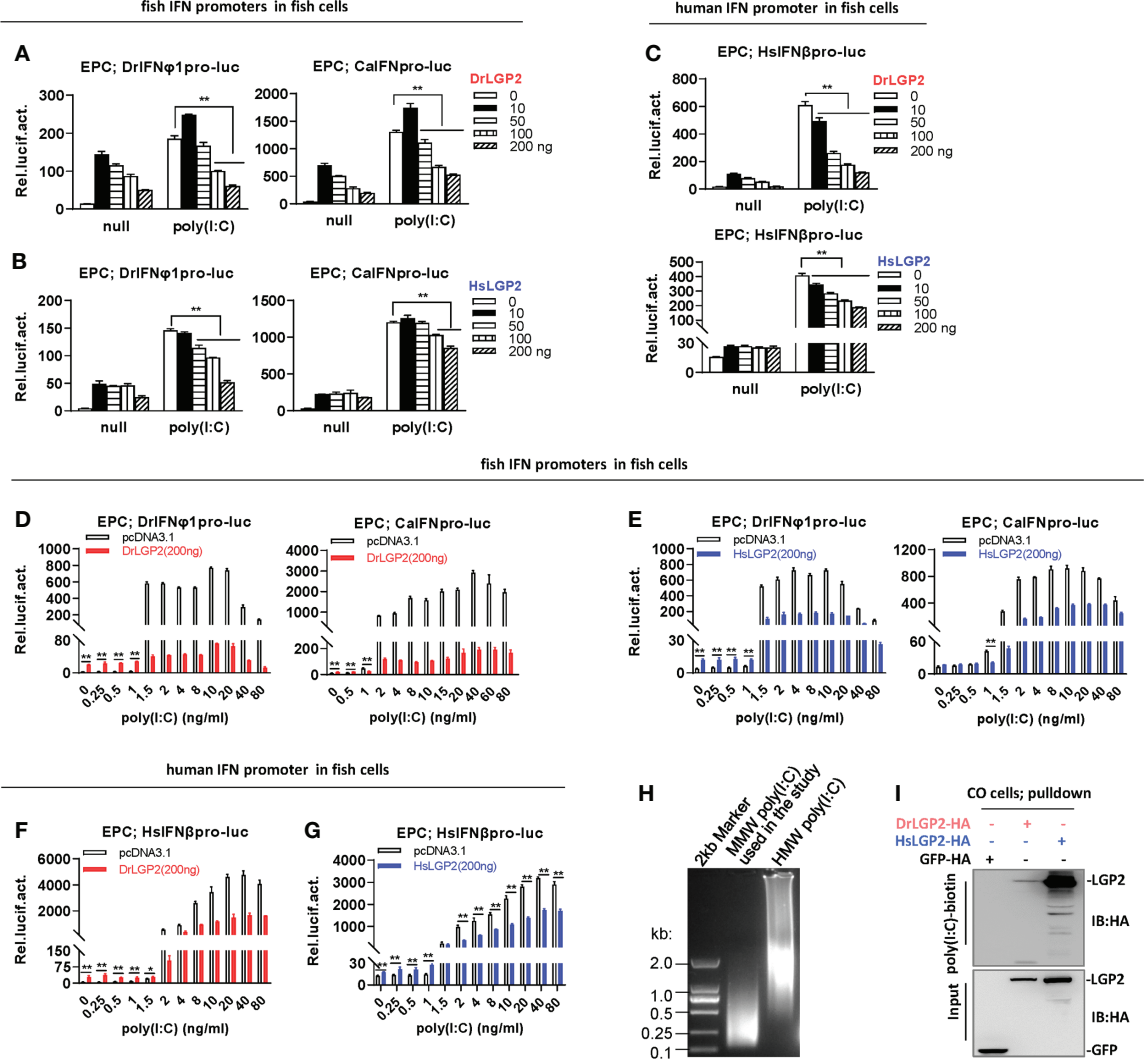
Zebrafish and human LGP2s play antithetic roles in regulating IFN response by poly(I:C) in fish cells **(A–C)** DrLGP2 and HsLGP2 downregulated fish IFN and human IFN promoter activation by poly(I:C) at a high concentration of 1 μg/ml in EPC cells. EPC cells seeded in 24-wells plates were co-transfected with DrIFNφ1pro-luc or CaIFNpro-luc **(A, B)**, or HsIFNβpro-luc (200 ng each) **(C)**, together with DrLGP2 or HsLGP2 at increasing doses (0, 10, 50, 100, 200 ng). 24 h later, cells were transfected with 1 μg/ml poly(I:C) for another 24 h, followed by luciferase assays. *P* values were calculated using ANOVA. **P < 0.01,*P < 0.05. **(D–G)** DrLGP2 and HsLGP2 switched a first positive role to a following negative one in regulating IFN response by increasing concentrations of poly(I:C) in EPC cells. EPC cells seeded overnight in 24-wells plates were co-transfected with DrIFNφ1pro-luc or CaIFNpro-luc **(D, E)**, or HsIFNβpro-luc **(F, G)**, together with DrLGP2 **(D, F)** or HsLGP2 **(E, G)** (200 ng each). 24 h later, cells were transfected with poly(I:C) at increasing doses for another 24 h, followed by luciferase assays. *P* values were calculated using Student’s t-test. **P<0.01. **(H)** Agarose electrophoresis showed the molecular weight spanning of MMW poly(I:C) and HMW poly(I:C). **(I)** RNA pull-down assays verified the binding of poly(I:C) to DrLGP2 and HsLGP2 in fish cells. CO cells seeded in 10 cm dishes were transfected with DrLGP2-HA, HsLGP2-HA or GFP-HA as control. 24 h later, cells were lysed. One-tenth of cell lysates were taken as input, the remaining was incubated with 100 ng biotinylated poly(I:C), followed by western blots with anti-HA antibody.

Luciferase assays were used to further determine the role of LGP2 in poly(I:C)-triggered IFN response. Similar to previous results (29), whereas overexpression of DrLGP2 alone provokes a strong IFN response at a low dose (10 ng) but gradually weak ones at high doses (>10 ng) in EPC cells, overexpression of DrLGP2 dose-dependently downregulated two fish promoter activation by poly(I:C) at a relatively high concentration (1 μg/ml) (**Figure 3A**). Similar inhibitory roles were seen for HsLGP2 (**Figure 3B**). Human IFNβ promoter activation by 1 μg/ml of poly(I:C) was also downregulated by either DrLGP2 or HsLGP2 (**Figure 3C**). Overexpression of DrLGP2 or HsLGP2 in another fish cells (CO) obtained the same results (**Supplementary Figures 2A–C**). These results indicated a negative role of LGP2 in fish cells responsive to poly(I:C) at a relatively high concentration.

However, titration of poly(I:C) in fish cells revealed antithetic roles of LGP2 responding to different concentrations of poly(I:C). It showed that transfection of EPC cells with poly(I:C) alone from 0.25 to 80 ng/ml resulted in a dose-dependent fish promoter activation, and co-transfection of DrLGP2 (200 ng) enhanced fish promoter activation by poly(I:C) at lower concentrations (<1 ng/ml), but inhibited the activation by poly(I:C) at higher concentration (>1 ng/ml) (**Figure 3D**), which was replicated when HsLGP2 was transfected instead of DrLGP2 (**Figure 3E**). Similarly, low concentrations (<1 ng/ml) of poly(I:C)-triggered HsIFNβ promoter activation was synergistically promoted, but high concentrations (>2 ng/ml) of poly(I:C)-triggered activation was significantly inhibited by DrLGP2 (**Figure 3F**) or HsLGP2 (**Figure 3G**). The replicated assays in CO cells obtained the same results (**Supplementary Figures 2D–G**). In addition, microscopy observation showed a nearly similar transfection efficiency of a GFP-Flag plasmid in CO cells when poly(I:C) was present at a low concentration (80 ng/ml) and a high concentration (1000 ng/ml) (**Supplementary Figure 2H**). Therefore, zebrafish and human LGP2s play opposing roles in regulating poly(I:C)-triggered IFN response in fish cells relative to the transfected concentrations of poly(I:C).

It has been established that in mammalian cells, synthetic poly(I:C) with >1 kb in size preferentially activates MDA5-dependent IFN expression, but can be converted to a RIG-I ligand by shortening length (45). The poly(I:C) used in the study was a MMW (medium molecular weight) poly(I:C) with molecular weights mostly between 0.1-2kb (**Figure 3H**), indicating that the MMW poly(I:C) can efficiently activate RIG-I signaling and also MDA5 signaling in mammalian cells. Consistently, DrLGP2 and HsLGP2 displayed a binding affinity to poly(I:C) in fish cells (CO cells) by RNA pulldown (**Figure 3I**).

### Zebrafish and human LGP2s play a negative role in regulating IFN response by high concentrations of poly(I:C) in mammalian cells

**Figure 4:**
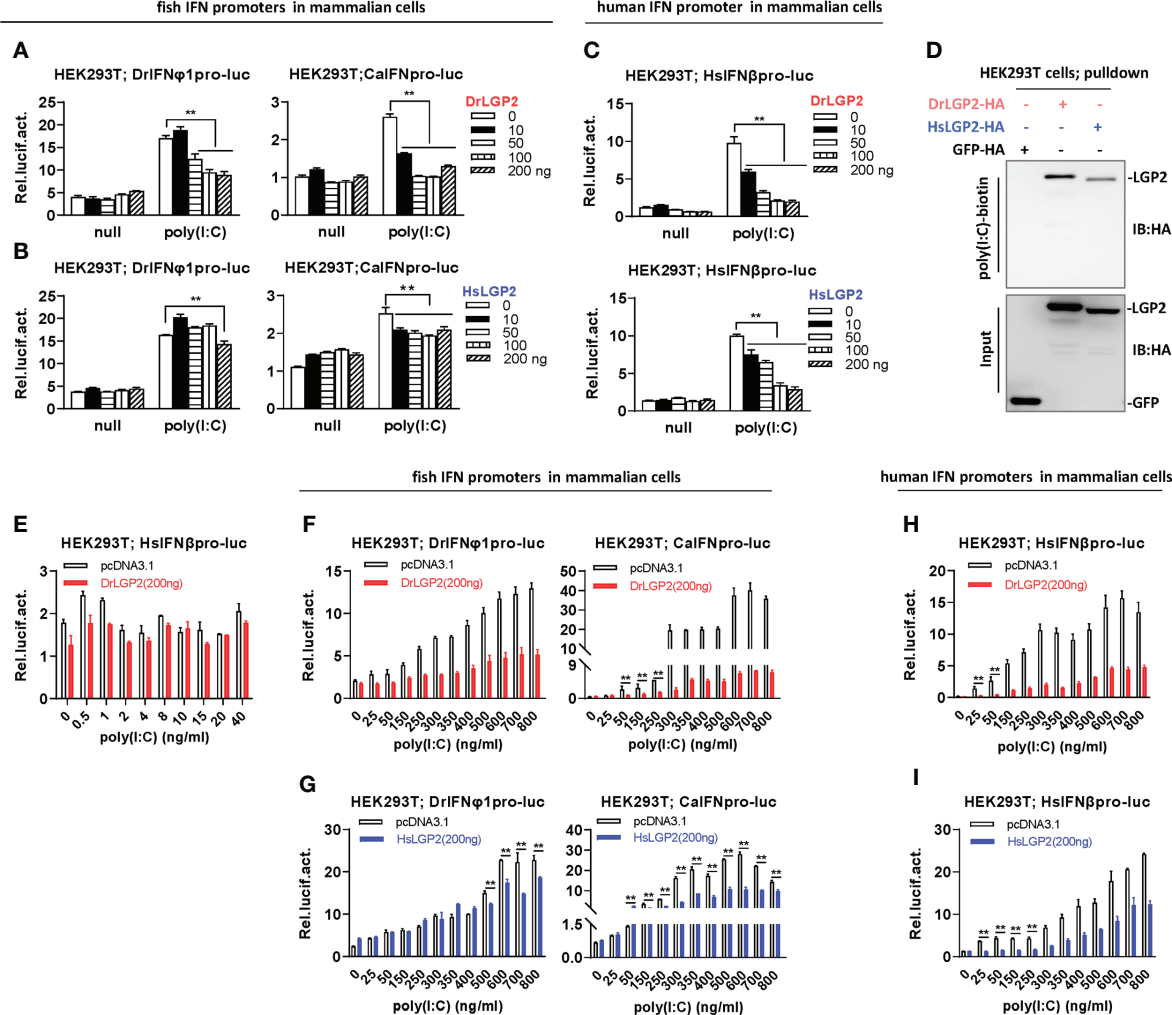
Zebrafish and human LGP2s play a negative role in regulating IFN response by high concentrations of poly(I:C) in mammalian cells **(A–C)** DrLGP2 and HsLGP2 downregulated fish IFN and human IFNβ promoter activation by poly(I:C) at a high concentration of 2 μg/ml in mammalian cells. HEK293T cells seeded in 24-wells plates were co-transfected with DrIFNφ1pro-luc or CaIFNpro-luc **(A, B)**, or HsIFNβpro-luc (200 ng each) **(C)**, together with DrLGP2 or HsLGP2 at increasing doses (0, 10, 50, 100, 200 ng). 24 h later, cells were transfected with 2 μg/ml poly(I:C) for another 24 h, followed by luciferase assays. *P* values were calculated using ANOVA. **P < 0.01. **(D)** RNA pull-down assays verified the binding of poly(I:C) to DrLGP2 and HsLGP2 in mammalian cells. HEK293T cells seeded in 10 cm dishes were transfected with DrLGP2-HA, HsLGP2-HA or GFP-HA as control. 24 h later, cells were lysed. One-tenth of cell lysates were taken as input, the remaining was incubated with 100 ng biotinylated poly(I:C), followed by western blots with anti-HA antibody. **(E–I)** Titration of poly(I:C) revealed a negative regulation of zebrafish and human LGP2s on IFN response in mammalian cells. HEK293T cells seeded in 24-wells plates were co-transfected with HsIFNβpro-luc **(E, H, I)**, or with DrIFNφ1pro-luc or CaIFNpro-luc **(F, G)**, together with DrLGP2 **(E, F, H)** or HsLGP2 **(G, I)** (200 ng each). Renilla vector (pRL-TK, 0.2 ng) was transfected as internal control. 24 h later, cells were transfected with poly(I:C) at increasing doses for another 24 h, followed by luciferase assays. *P* values were calculated using Student’s t-test. **P < 0.01.

Similar to aforementioned results, either low or high doses of DrLGP2 alone did not activate fish IFN promoters in mammalian cell lines (**Figures 4A–C**). A high concentration of poly(I:C) (2 μg/ml) could trigger a significant activation of two fish promoters in HEK293T cells; however, this activation was dose-dependently downregulated by DrLGP2 (**Figure 4A**), and also by HsLGP2 (**Figure 4B**). This was true for poly(I:C) (2 μg/ml)-mediated HsIFNβ promoter activation in HEK293T cells (**Figure 4C**). Similar results were replicated in COS7 (**Supplementary Figures 3A–C**). Consistently, either DrLGP2 or HsLGP2 displayed a binding affinity to poly(I:C) in HEK293T cells (**Figure 4D**).

Unlike that IFN promoters were easily activated by poly(I:C) at very low concentrations in fish cells (<1 ng/ml) (**Figures 3D–G**), titration of poly(I:C) from 0.5 to 40 ng/ml in HEK293T cells did not yield obvious stimulatory effects on HsIFNβ promoter activation (**Figure 4E**). Thus, the concentrations of poly(I:C) from 25 to 800 ng/ml were used for following titration experiments in mammalian cells. Under these conditions, fish IFN promoters were dose-dependently activated in HEK293T cells; however, this activation was not synergistically promoted but significantly inhibited by DrLGP2 (**Figure 4F**) or HsLGP2 (**Figure 4G**). The same inhibition was observed for poly(I:C)-mediated human IFNβ promoter activation by DrLGP2 (**Figure 4H**) or HsLGP2 (**Figure 4I**). We replicated these assays in COS7 cells and obtained the same results (**Supplementary Figures 3D–G**). It is noted that <800 ng/ml of poly(I:C) was transfected in these assays, although microscopy observation showed a marginal reduction of transfection efficiency of GFP-Flag plasmid in HEK293T cells when transfected with poly(I:C) at 2000 ng/ml, as compared to transfection of poly(I:C) at 800 ng/ml (**Supplementary Figure 3H**). These data suggest that, in mammalian cells transfected with poly(I:C) at relatively high concentrations from 25 ng/ml to 800 ng/ml, DrLGP2 and HsLGP2 exert an inhibitory regulation on poly(I:C)-induced IFN response.

### Zebrafish and human LGP2s play antithetic roles under low concentrations of poly(I:C) in mammalian cells and do so alone in fish cells

**Figure 5:**
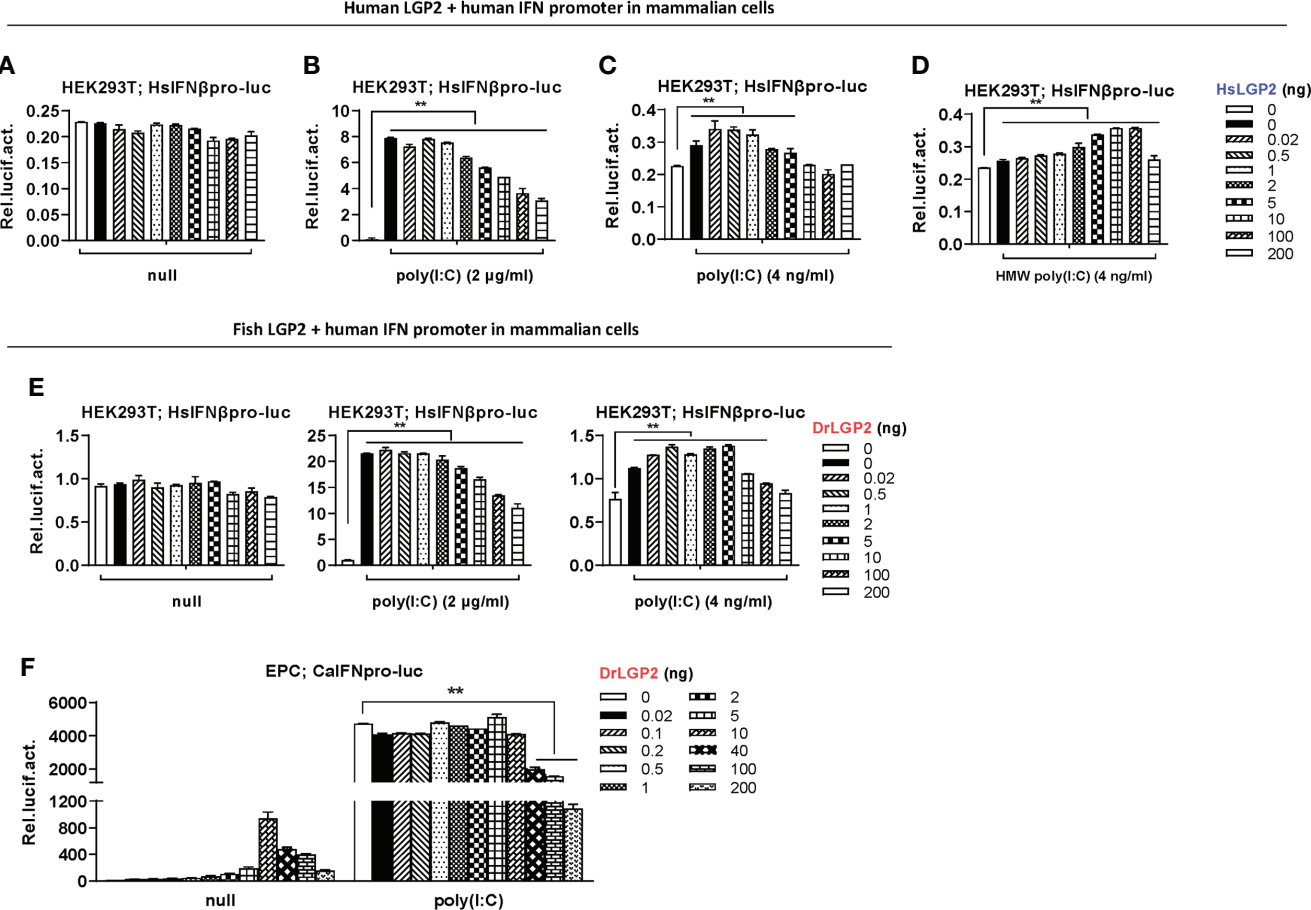
Zebrafish and human LGP2s play antithetic roles under low concentrations of poly(I:C) in mammalian cells and do so alone in fish cells. **(A–E)** Zebrafish and human LGP2s played antithetic roles under low concentrations of poly(I:C) in mammalian cells. HEK293T cells seeded in 24-wells plates were co-transfected with HsIFNβpro-luc (200ng), together with HsLGP2 **(A–D)** or DrLGP2 **(E)** (200 ng each). 24h later, cells were transfected again with MMW poly(I:C) [indicated as poly(I:C) in the text or all Figures] at 2 μg/ml **(B, E)** or at 4 ng/ml **(C)**, or with HMW poly(I:C) at 4 ng/ml **(D)**. Renilla vector (pRL-TK, 0.2 ng) was transfected as internal control. Another 24 h later, cells were collected for luciferase assays. *P* values were calculated using ANOVA. **P < 0.01. **(F)** Overexpression of zebrafish or human LGP2s alone revealed antithetic roles in mammalian cells. EPC cells seeded in 24-well plates were transfected with DrLGP2 at the indicated increasing doses for 48 h. Or at 24 h post transfection, cells were transfected again with poly(I:C) at a high concentration of 1 μg/ml for another 24 h, followed by luciferase assays. *P* values were calculated using ANOVA. **P < 0.01.

Since mammalian cells could not efficiently respond to transfection of LGP2 alone (**Figure 2F**) or poly(I:C) at low concentrations (**Figure 4E**), it is high of interest to wonder LGP2 functions in mammalian cells responsive to poly(I:C) at relatively low concentrations. As expectedly, titration of HsLGP2 alone from 0.02 to 200 ng in HEK293T cells did not lead to human IFNβ promoter activation (**Figure 5A**), and a high concentration of poly(I:C) (2 μg/ml)-triggered human IFNβ promoter activation was inhibited by HsLGP2 in a dose-dependent manner (**Figure 5B**). Interestingly, when poly(I:C) was transfected at a low concentration (4 ng/ml), low dose of human LGP2 (<1 ng) resulted in a statistically-significant human IFNβ promoter activation in a dose-dependent manner, and the peak activation was gradually weakened along with the dose of HsLGP2 (>1 ng) (**Figure 5C**). Similarly, when HEK293T cells were transfected by 4 ng/ml of HMW poly(I:C), a second poly(I:C) indicated in **Figure 3H**, HsLGP2-triggered antithetic regulation was observed (**Figure 5D**). Titration of DrLGP2 also showed an exclusively dose-dependently inhibition of human IFNβ promoter activation by poly(I:C) at a high concentration (2 μg/ml) (middle panel in **Figure 5E**), but a stable antithetic regulation was detected in the presence of poly(I:C) at a low concentration (4 ng/ml) (right panel in **Figure 5E**). These results clearly indicated that, in mammalian cells when transfected with poly(I:C) at low concentrations, overexpression of LGP2 induces an IFN response with weak strength but statistical significance, further implying that zebrafish and human LGP2s exert a positive regulation of IFN response at a low dose and a negative one at a high dose.

Since fish cells could efficiently respond to transfection of LGP2 alone (**Figure 2E**) or poly(I:C) at low concentrations (**Figures 3D–G**), we speculated that DrLGP2 alone could act in fish cells, as did HsLGP2 in mammalian cells under poly(I:C) at low concentrations. Similar to our previous results (36), titration of DrLGP2 in fish cells showed that, low doses of DrLGP2 alone (≤10 ng) dose-dependently activated fish IFN promoter, up to a peak when 10 ng of DrLGP2 was transfected, which was in turn decreased gradually along with DrLGP2 doses increasing (>10 ng) (**Figure 5F**). These results indicated that there was a DrLGP2 self-mediated inhibition in fish cells when DrLGP2 was overexpressed alone. That is, the highest IFN promoter activation at 10 ng of DrLGP2 was dose-dependently diminished by extra DrLGP2. Moreover, a high concentration of poly(I:C) (1μg/ml)-triggered IFN promoter activation was impaired by DrLGP2, particularly at high doses (>10 ng) (**Figure 5F**), as did HsLGP2 in mammalian cells (**Figure 5B**). Therefore, zebrafish and human LGP2s act similarly in both fish cells and mammalian cells, playing antithetic roles in regulating IFN response. The different sensitivity to poly(I:C) between fish cells and mammalian cells might account for the finding that overexpression of zebrafish or human LGP2 alone directly activates IFN response in fish cells but not in mammalian cells.

### Zebrafish and human LGP2s play antithetic roles in regulating IFN response by MDA5/RIG-I in fish cells

**Figure 6:**
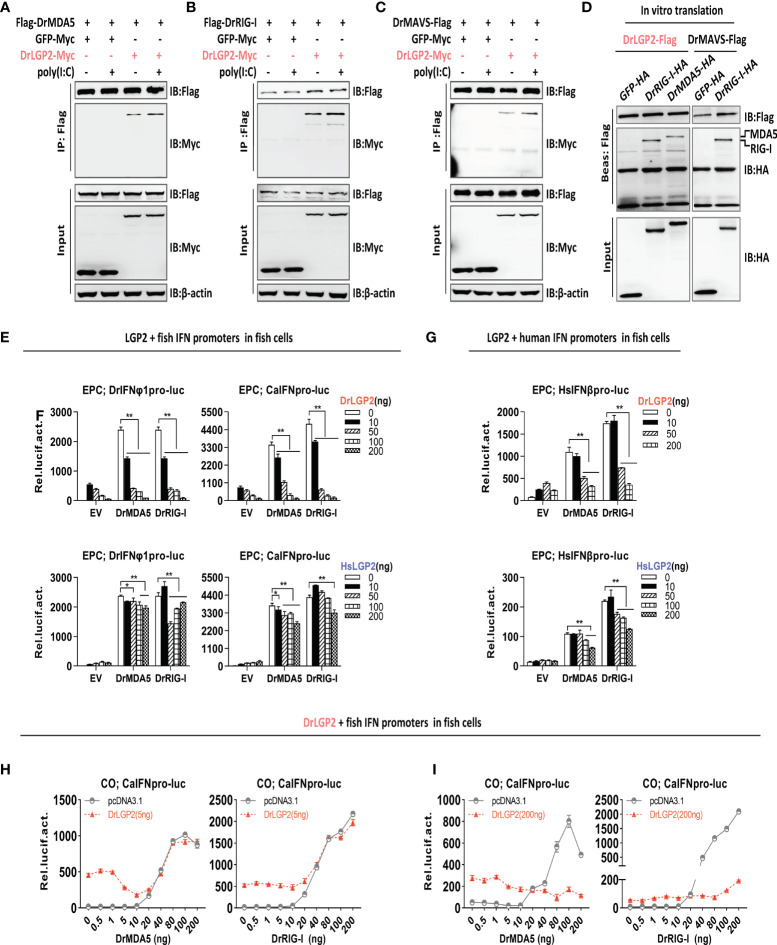
Zebrafish and human LGP2s play antithetic roles in regulating MDA5-/RIG-I-triggered IFN response in fish cells dependently of the exact doses of MDA5/RIG-I **(A–C)** Zebrafish LGP2 bound to DrMDA5 **(A)**, DrRIG-I **(B)** or DrMAVS **(C)** independently of poly(I:C) by Co-IP assays. HEK293T cells seeded in 10 cm dishes overnight were transfected with LGP2-myc, together with Flag-DrMDA5 **(A)**, Flag-DrRIG-I **(B)**, DrMAVS-Flag **(C)** (5 μg each), in the presence or absence of poly(I:C) at 200 ng/ml) **(A–C)**. Cell lysates were immunoprecipitated with anti-Flag Ab, followed by western blot analysis of the immunoprecipitates with anti-myc Ab. **(D)** *In vitro*-translated DrLGP2 bound to *in vitro*-translated DrMDA5 and DrRIG-I by pull-down assays. 50 μl of *in vitro*-translated DrLGP2-Flag was incubated with *in vitro*-translated DrMDA5-HA or *in vitro*-translated DrRIG-I-HA in NP40 lysis buffer at 4°C for 12 h. The bead-bound protein complex was performed by western blotting analysis with anti-Tag Ab. *In vitro*-translated DrMAVS-Flag was incubated with *in vitro*-translated DrRIG-I-HA as positive control, and with GFP-HA as negative control. **(E–G)** DrLGP2 and HsLGP2 downregulated IFN promoter activation in fish cells induced by DrMAD5 or DrRIG-I at a high dose. EPC cells seeded in 24-wells plates were transfected with DrIFNφ1pro-luc or CaIFNpro-luc **(E, F)** or HsIFNβpro-luc **(G)**, DrMAD5 or DrRIG-I (200 ng each), DrLGP2 or HsLGP2 at increasing doses (0, 10, 50, 100, 200 ng) for 24 h, followed by luciferase assays. *P* values were calculated using ANOVA. **P<0.01, *P<0.05. **(H, I)** Titration of DrMDA5 and DrRIG-I revealed DrLGP2-mediated antithetic regulation of IFN response in fish cells. CO cells seeded in 24-well plates were transfected with CaIFNpro-luc (200 ng), DrLGP2 at 5 ng **(H)** or at 200 ng **(I)**, DrMDA5 or DrRIG-I at increasing doses for 24 h, and finally collected for luciferase assays.

In mammals, titration of LGP2 expression suggests that low levels of LGP2 synergize with MDA5 as a positive regulator, but high levels of LGP2 act as an inhibitor of RIG-I and MDA5 signaling (7, 15, 16). We further attempted to investigate LGP2-mediated regulation on RIG-I- and MDA5-triggered IFN signaling in fish cells. Initially, Co-IP assays revealed the interactions of DrLGP2 with DrMDA5 (**Figure 6A**), DrRIG-I (**Figure 6B**), and DrMAVS (**Figure 6C**), in the presence or absence of poly(I:C). This binding was not enhanced along with increasing concentrations of poly(I:C) (**Supplementary Figure 4A**). Using *in-vitro* translated proteins, pull-down assays verified that LGP2 directly bound to either zebrafish MDA5 or RIG-I (**Figure 6D**).

Subsequently, luciferase assays showed that overexpression of either DrMDA5 or DrRIG-I at a high dose (200 ng) directly activated fish promoter activation in EPC cells, which was significantly blocked by DrLGP2 (**Figure 6E**), or by HsLGP2, albeit to a weak degree (**Figure 6F**). The same inhibition for HsIFNβ promoter activation was observed (**Figure 6G**), which was further supported by RT-PCR analysis of cellular *ifn* and ISG (*mx, viperin, irf3* and *irf7*) expression in EPC cells under the same conditions (**Supplementary Figure 4B**).

Finally, titration of DrRIG-I/DrMDA5 showed that DrLGP2, at either a low or high dose (5 ng versus 200 ng), displayed a synergistical regulation of IFN signaling triggered by DrMDA5/DrRIG-I at low doses (DrMDA5: <40 ng; DrRIG-I: <20 ng) **Figures 6H, I**), particularly for 5 ng of LGP2 (**Figure 6H**), but an inhibitory regulation of IFN signaling by DrMDA5/DrRIG-I at high doses (≥40 ng) (**Figure 6H, I**), particularly for 200 ng of LGP2 (**Figure 6I**). These results indicated that LGP2 plays antithetic regulation in fish RIG-I/MDA5-triggered IFN signaling dependently of the exact doses of DrMDA5/DrRIG-I in fish cells.

### Zebrafish and human LGP2s promote MDA5 signaling under low concentrations of poly(I:C) and downregulate RIG-I/MDA5 signaling mainly under high concentrations of poly(I:C) in mammalian cells

**Figure 7:**
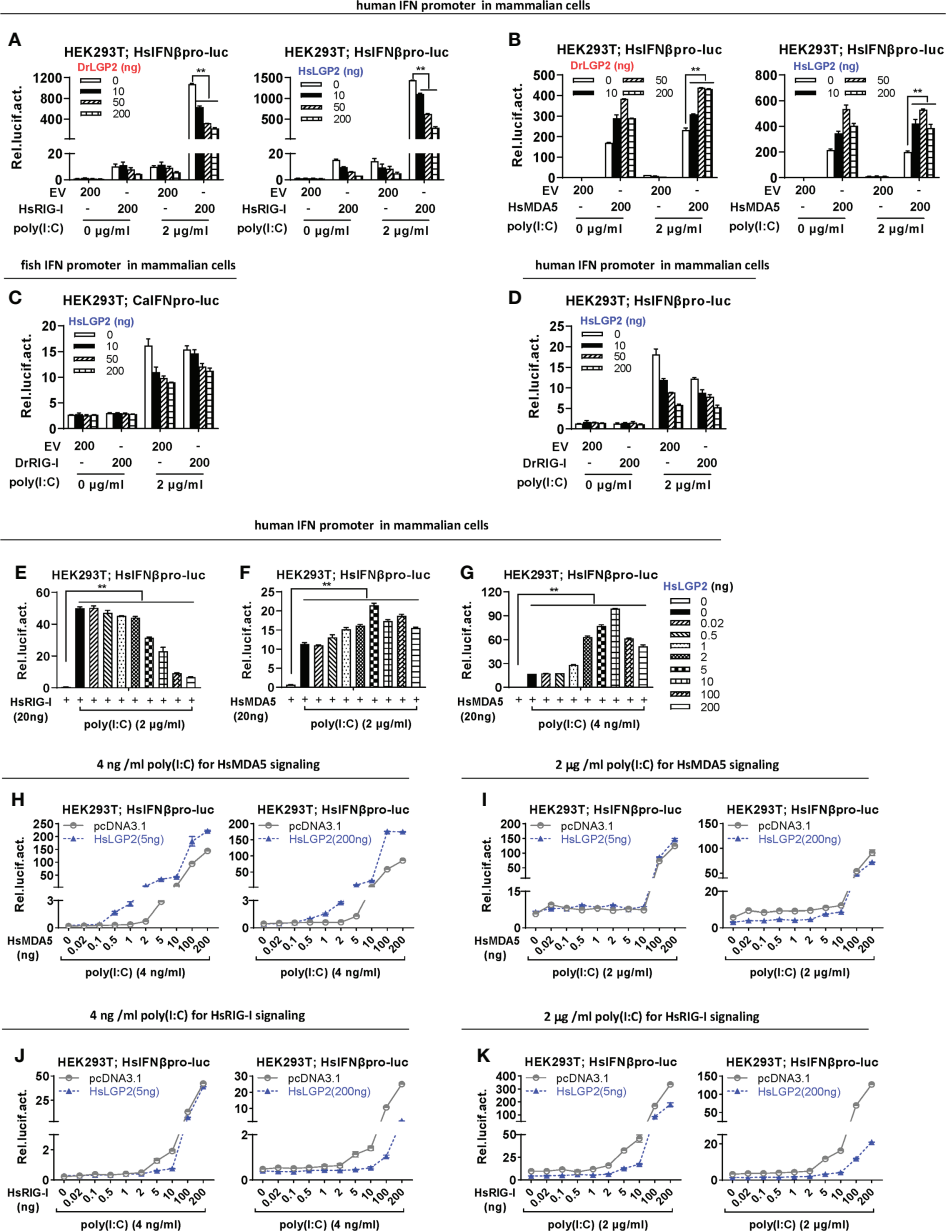
Zebrafish and human LGP2s promote MDA5 signaling under low concentrations of poly(I:C) and downregulate RIG-I/MDA5 signaling mainly under high concentrations of poly(I:C) in mammalian cells **(A, B)** DrLGP2 and HsLGP2 promoted human IFNβ activation by HsMDA5 at a high dose and downregulated IFN response by HsRIG-I at a high dose in mammalian cells. HEK293T cells seeded in 24-wells plates were transfected with HsIFNβpro-luc, DrLGP2 or HsLGP2 at increasing doses, together with HsRIG-I **(A)** or HsMAD5 **(B)** (200 ng each). 24 h later, cells were transfected again with or without poly(I:C) (2 μg/ml) for another 24 h, followed by luciferase assays. *P* values were calculated using ANOVA. **P<0.01. **(C, D)** HsLGP2 negatively regulated zebrafish RIG-I signaling in mammalian cells. HEK293T cells seeded in 24-wells plates were transfected as in A with the indicated plasmids. **(E–G)** Titration of HsLGP2 expression revealed differential regulation of RIG-I- and MDA5-triggered IFN signaling by HsLGP2 in the presence of poly(I:C) in mammalian cells. HEK293T cells seeded in 24-wells plates were transfected with HsIFNβpro-luc, HsLGP2 at increasing doses, together with HsRIG-I **(E)** or HsMAD5 **(F, G)** (200 ng each). 24 h later, cells were transfected with or without poly (I:C) at 2 μg/ml **(E, F)** or at 4 ng/ml **(G)** for another 24 h, followed by luciferase assays. *P* values were calculated using ANOVA. **P<0.01. **(H–K)** Titration of HsMDA5/MsRIG-I showed that HsLGP2 promoted HsMDA5 signaling under low concentrations of poly (I:C) and downregulate HsRIG-I signaling mainly under high concentrations of poly (I:C) in mammalian cells. HEK293T cells seeded in 24-wells plates were transfected with HsIFNβpro-luc, HsLGP2, together with increasing doses of HsMDA5 **(H, K)** or HsRIG-I **(J, K)**. 24 h later, cells were transfected with or without poly (I:C) at 4 ng/ml **(H, J)** or 2 μg/ml **(I K)** for another 24 h, followed by luciferase assays.

We next investigated LGP2-mediated regulation on RIG-I- and MDA5-triggered IFN signaling in mammalian cells. Similar to the findings in mammals (3, 8, 14, 15, 21, 26, 27), transfection of HsRIG-I alone resulted in a weak activation of human IFNβ promoter but a robustly enhanced one in HEK293T cells when transfected together with poly(I:C) at 2 μg/ml, a relatively high concentration (**Figure 7A**). However, overexpression of MDA5 sometimes gave a nearly similar IFNβ promoter activation in the presence and absence of poly(I:C) (**Figure 7B**). HsRIG-I-triggered human promoter activation was dose-dependently inhibited by either DrLGP2 or HsLGP2 (**Figure 7A**), but HsMDA5-triggered promoter activation was mostly promoted by either DrLGP2 or HsLGP2 (**Figure 7B**), which was replicable with fish IFN promoters (**Supplementary Figures 5A, B**). Similarly, DrRIG-I also required poly(I:C) stimulation in HEK293T cells to acquire the full potential to activate fish IFN promoter (**Figure 7C**) and human IFNβ promoter (**Figure 7D**), which could be inhibited by HsLGP2.

Subsequent titration assays were performed to further determine LGP2-mediated regulation on RIG-I/MDA5 signaling in HEK293T cells under poly(I:C) transfection. In the presence of poly(I:C) at a high concentration (2 μg/ml), titration of HsLGP2 showed a dose-dependently inhibition on RIG-I-triggered promoter activation (**Figure 7E**), and a fluctuating promotion on MDA5-triggered promoter activation, which was gradually enhanced by low doses of HsLGP2 and thereafter attenuated by high doses of HsLGP2 (**Figure 7F**). Notably, HsLGP2 gave a robustly fluctuating promotion on HsMDA5 signaling when poly(I:C) was present at 4 ng/ml instead of 2 μg/ml (**Figure 7G**), with a pattern similar to titration of HsLGP2 under low concentrations of poly(I:C) (**Figure 5C**), indicating that HsLGP2 exerts a positive regulation of HsMDA5-triggered IFN response at a low dose and a negative one at a high dose. These results above could be replicated in HEK293T cells when HsLGP2 was replaced by DrLGP2 (**Supplementary Figure 5C**) or when HsRIG-I/HsMDA5 were replaced by DrRIG-I/DrMDA5 (**Supplementary Figures 5D, E**). Given that the concentrations of poly(I:C) might be analogous to the concentrations of intracellular dsRNA during viral infection, we next compared HsLGP2-mediated regulation on HsMDA5/HsRIG-I signaling in HEK293T cells under poly(I:C) at 4 ng/ml and 2 μg/ml.

In the presence of poly(I:C) at 4 ng/ml, titration of HsMDA5 from 0.5 to 200 ng showed that HsLGP2, at either a low dose or a high dose (5 ng versus 200 ng), displayed a stably synergistical regulation of HsMDA5-triggered IFN signaling (**Figure 7H**). However, in the presence of poly(I:C) at 2 μg/ml, we did not detect the synergistical regulation by HsLGP2 at either 2 ng or 200 ng (**Figure 7I**). Particularly when HsMDA5 dose was <10 ng, 200 ng of HsLGP2 exhibited a negative regulation (**Figure 7I**, right panel). These results indicated that HsLGP2 promotes HsMDA5 signaling mainly in the presence of poly(I:C) at 4 ng/ml (analogous to a low dose of viral dsRNA) and instead, a high dose (200 ng) of LGP2 exerts a negative regulation of HsMDA5 signaling in the presence of poly(I:C) at 2 μg/ml (analogous to a high dose of viral dsRNA).

Similar titration of HsRIG-I showed that, in the presence of poly(I:C) at 4 ng/ml, HsLGP2 displayed an inhibitory regulation of HsRIG-I signaling, with a relatively narrow dose window of HsRIG-I initially from 5 ng (**Figure 7J**). Particularly when 5 ng of HsLGP2 was used, only 5 ng and 10 ng of HsRIG-I-directed IFN signaling were inhibited (**Figure 7J**, left panel). In the presence of poly(I:C) at 2 μg/ml, HsLGP2 gave a significant inhibition of HsRIG-I signaling, particularly at 200 ng and with a wide dose window of HsRIG-I initially from 0.02 ng (**Figure 7K**). These results indicated that HsLGP2 inhibits HsRIG-I signaling mainly in the presence of poly(I:C) at 2 μg/ml.

## Discussion

Using the same overexpression assays, it has been shown that fish LGP2 efficiently induces IFN response in fish cells, but this is not the case for human LGP2 in mammalian cells (3, 5, 29, 36). Given structure conservation of fish and human LGP2 proteins, these differences promoted us to compare their function differences in fish cells and mammalian cells. Our data clearly indicate that overexpression of zebrafish or human LGP2 directly stimulates IFN response in fish cells but not in mammalian cells, implying that there should be certain unknown disparities between fish cells and mammalian cells resulting in the observed differences. We subsequently found that the same happens to RIG-I. In fish cells, overexpression of zebrafish RIG-I exhibits a direct potential to stimulate IFN response in fish cells, but in mammalian cells, it requires supplementary stimulation of poly(I:C) to arouse the full potential, as does human RIG-I in mammalian cells (3, 26, 27, 46). It is well documented that human RIG-I is fully activated only under viral infection or poly(I:C) transfection, because it keeps a self-inhibition conformation in resting mammalian cells (1). If this interpretation is reasonable for the differential behaviors of LGP2 in fish cells and mammalian cells, the different sensitivity of fish cells and mammalian cells to poly(I:C), a synthetic analog of dsRNA, might account for the finding that overexpression of zebrafish or human LGP2 alone can directly activate IFN response in fish cells but not in mammalian cells.

Luciferase assay is hypersensitive to modulate the subtle regulation of gene expression. Titration assays were widely used in the current study, and consistent results could be obtained when total doses of transfected plasmids were used at less than a threshold, such as <600 ng in 0.5 ml/well in 24-well plates. Under these conditions, marginal cytotoxicity, if occurred at high concentrations of transfected plasmids or poly(I:C) (**Supplemental Figure 3H**), did not confound the consistent results due to in parallel transfection of Renilla plasmid pRL-TK as internal control, which guarantees the reproducibility of results in luciferase assays. The data in the present study suggest that fish cells are more sensitive to poly(I:C) transfection than mammalian cells. Whereas <1 ng/ml of poly(I:C) is able to efficiently activate IFN promoters in fish cells (**Figures 3D–G**), less than 40 ng/ml of poly(I:C) cannot stably do so in mammalian cells (**Figure 4E**). These differences might be an essential factor directly contributing to the differential results that ectopically-expressed LGP2 alone has a stimulatory potential to IFN promoter activation in fish cells but not in mammalian cells. It is possible that a trace amount of dsRNA, which has been produced during plasmid transfection, is sufficient to facilitate LGP2 (or RIG-I)-mediated promoter activation in fish cells, but this is not enough in mammalian cells, thus requiring additional transfection of poly(I:C). Interestingly, luciferase assays have shown that LGP2 triggered IFN response in mammalian cells only under low concentrations (4 ng/ml) of poly(I:C), at which poly(I:C) has no or marginal effects on IFN promoter activation; and at a high concentration (2 μg/ml), poly(I:C) transfection induces high levels of IFN promoter activation, which is invariably inhibited by LGP2 (**Figure 5**). Despite weak strength, the stimulatory potential of LGP2 under low concentrations of poly(I:C) in mammalian cells is easily detectable by titration of LGP2, highlighting that the stimulatory potential of LGP2 under this condition is not the result of experimental errors and should not be ignored. These results also suggest that the LGP2’s stimulatory pattern in mammalian cells under low concentrations of poly(I:C) is indeed similar to that in fish cells by titration of LGP2 alone without poly(I:C) (**Figure 5F**). That is, if the stimulatory potential of ectopically-expressed LGP2s in fish cells requires pre-conjugation to trace dsRNA produced by plasmid transfection, as does human LGP2 in mammalian cells (15–17, 47), it is easy to understand that fish and human LGP2s actually display a same regulatory effect on stimulating IFN response, in fish cells by overexpression alone, and in mammalian cells by combined transfection of poly(I:C) at low concentrations (**Figure 5**).

Poly(I:C) transfection is easily titrated to qualitatively mimic the intracellular dsRNA during virus infection. Given that virus infection produces increasing amounts of viral dsRNA in cells (48), it is reasonable that low concentrations (such as 4 ng/ml) of poly(I:C) might be analogue to the intracellular concentrations of viral RNA at the early stage of virus infection and high concentrations correspond to the late stage of virus infection. Similar to our previous results (29), titration of poly(I:C) in fish cells reveals that zebrafish LGP2, either at a low dose or at a high dose, synergistically promotes IFN response by low concentrations of poly(I:C), but significantly inhibits IFN response by high concentrations of poly(I:C), indicating that zebrafish LGP2 might act as an essential activator of IFN response at the early phase of virus infection, but as a negative regulator at the late phase of viral infection, which has been verified by SVCV infection in fish cells (29). In mammalian cells, titration of poly(I:C) does not replicate the similar dual roles of LGP2; however, the findings that LGP2 promotes IFN promoter activation at low concentrations of poly(I:C) and inhibits IFN promoter activation at high concentrations of poly(I:C) strongly indicate that LGP2 indeed plays dual roles in regulating IFN response in mammalian cells, similar to that in fish cells. A widely-accepted viewpoint in mammals is that LGP2 synergies with MDA5 to exert positive regulation of IFN response (7, 14–16, 21, 49). Therefore, we speculate that the basally-expressed MDA5 activation, in fish cells by the trace dsRNA produced during plasmids transfection and in mammalian cells by transfection of a low concentration of poly(I:C), might be necessary for the stimulatory potential of LGP2 when it is overexpressed alone. This should be true, because under poly(I:C) transfection, titration of LGP2 in mammalian cells yields a similar regulatory pattern in the absence or presence of MDA5, but with a stronger stimulatory ability in the presence of MDA5 than in the absence of MDA5 (**Figure 7G** versus **Figure 5C**).

The data in the current study further suggest that in mammalian cells, LGP2 promotes IFN response by synergistical enhancing MDA5 signaling in the presence of poly(I:C) at low concentrations, and inhibits IFN response by downregulating RIG-I signaling and MDA5 signaling in the presence of poly(I:C) at high concentrations. Firstly, human LGP2 mediates the best synergistic regulation under a low concentration of poly(I:C) (**Figures 7G** vs. **7F**). Secondly, RIG-I, MDA5 and LGP2 are, as typical ISGs, induced by virus infection, with a low expression level at the early stage of virus infection and a relatively high level at the late stage (3, 5, 29, 46). Titration of MDA5 expression showed either a low or high dose of human LGP2 elicits a significant and sustainable promotion on MDA5 signaling in mammalian cells when transfected by poly(I:C) only at a low concentration (4 ng/ml) and instead, LGP2-mediated inhibition is easily detected at a high concentration (2 μg/ml) of poly(I:C) particularly when LGP2 is transfected at high dose of 200 ng (**Figures 7I** vs. **7H**), thereby indicating that human LGP2 acts as an activator mainly at the early stage of viral infection. Thirdly, in the presence of poly(I:C) at 2 μg/ml, high doses of human LGP2 elicit a most significant inhibition of RIG-I signaling with a wide dose window of RIG-I; and in the presence of poly(I:C) at 4 ng/ml, human LGP2 downregulates RIG-I signaling with a very narrow dose window of RIG-I, particularly when LGP2 is expressed at a low dose (5 ng), strongly indicating that human LGP2 inhibits RIG-I signaling mainly at the late stage of virus infection.

In fish cells, titration of RIG-I and MDA5 shows that LGP2 promotes IFN response by low doses of RIG-I and MDA5 but inhibits IFN response by high doses of RIG-I and MDA5 (**Figures 6H, I**). Regardless of the existing differences between fish cells and mammalian cells, these results have revealed a function switch of LGP2 in both fish cells and mammalian cells responding to poly(I:C) or virus infection. Given that RIG-I and MDA5 can sense different dsRNA virus species in mammals (1), our results suggest that, when a certain RNA virus infection is sensed by both RIG-I and MDA5, it is possible that at the early stage of virus infection, LGP2 is expressed at low levels and thus mainly promotes MDA5 signaling to enhance host IFN response for virus clearance, and at the same time, LGP2 of low expression levels does not nearly inhibit RIG-I signaling. At the late stage of virus infection, LGP2 is induced up to high expression levels, which does not enhance MDA5 signaling anymore and instead inhibits RIG-I signaling, also MDA5 signaling, to balance host IFN response. Consistently, LGP2-mediated downregulation of MDA5 signaling is always detected when 200 ng of LGP2 is transfected under 2 μg/ml of poly(I:C) (**Figure 7I**, right panel), similar to previous studies in mammals (4, 5). In addition, if cells are infected with a virus that is only sensed by RIG-I, the positive role of LGP2 should not be neglected either. Recent findings have revealed that human LGP2 is essential for constitutive expression of *ifn* and ISGs dependently of the basally-expressed MDA5 (50, 51), which is necessary for rapid onset of IFN response toward virus infection (52–54).

Our notion might be helpful to interpret the controversial results in two LGP2-transgenic mice responsive to virus infection. Both transgenic mice exhibit better survival advantages than WT mice (11, 12). Surprisingly, they both have a diminished IFN response and a reduced viral load, one from 4 to 8 d post viral infection (12), and one from 8 to 11 d post infection (11). Based on our notions in this paper, the titers of viral replication and IFN expression detected by the authors should be the real-time state of both transgenic mice at the late stage of virus infection, probably indicating that LGP2 functions as a negative regulator of IFN response at this time. Although IFN response is not detected in both transgenic mice at the early phase of viral infection, a LGP2-deficient mouse actually displays less IFN production within 24 h post infection (9), supporting the notion that LGP2 functions as a positive regulator of IFN response at the early stage of virus infection.

The function switch of LGP2 is also illuminated in fish cells by titration of LGP2 alone (**Figure 5F**), and in mammalian cells by titration of LGP2 under low concentrations of poly(I:C) (**Figures 5C–E**), or under MDA5 and poly(I:C) together (**Figures 7F, G**). If we understand that the simultaneous transfection of poly(I:C) and/or MDA5 in mammalian cells is indeed a supplementary stimulation for LGP2’s regulatory potential, these results have shown that low doses of LGP2 yield a dose-dependently activation of IFN response, up to a peak that is dose-dependently weakened by the extra LGP2 itself. These results are previously explained by a dose-dependent biphasic switch model, emphasizing a positive regulation when LGP2 is present at low doses but a negative regulation when LGP2 is at high doses (7, 14–16). However, it is obvious that this model is hard to interpret why low doses of LGP2 harbor the ability to inhibit IFN response triggered by high amounts of poly(I:C) or/and RIG-I/MDA5 in fish cells and mammalian cells. If we think that the exact doses of IFN stimuli, including LGP2 itself at lower levels, are generally proportional to the resultant amounts of IFN products, these results imply that the function switch of zebrafish LGP2 might be tightly related to the expression levels of cellular IFN by IFN stimuli or LGP2 itself (29, 36). Despite lack of direct evidences, a reasonable interpretation is that at the early stage of viral infection, cellular IFN is expressed at low levels to enable a positive regulation of LGP2, and at the late stage of infection, cellular IFN is expressed to a threshold level that might drive LGP2 to a negative role.

In summary, the existing disparities of fish and human LGP2s in previous studies might be as a result of the difference in sensitivity of fish cells and mammalian cells to dsRNA. Given that zebrafish LGP2 promotes IFN response at the early stage of virus infection through MDA5 (36) and titration of poly(I:C) may be analogous to the intracellular concentrations of viral dsRNA at different stages of virus infection, our results provide *in vitro* evidences that LGP2 indeed plays a conserved dual function in fish cells and mammalian cells, as a activator of IFN response by promoting MDA5 signaling at the early stage of virus infection and an inhibitor by impairing RIG-I/MDA5 signaling at the late stage of virus infection.

## Data availability statement

The original contributions presented in the study are included in the article/**Supplementary Material**. Further inquiries can be directed to the corresponding author.

## Author contributions

Y-BZ and X-YG designed the research; X-YG performed the experiments; Y-BZ, X-YG, Z-LQ, Y-LL, H-YS, XZ, and CD. analyzed the data. J-FG provided useful insights and reagents; X-YG and Y-BZ wrote the paper. All authors have approved the final version.

## Funding

This work was supported by the Grants from the Strategic Priority Research Program of the Chinese Academy of Sciences (XDA24010308), the National Key R&D Program of China (2018YFD0900302), the National Natural Science Foundation (31772875 and 31972826), and the Application Fundamental Frontier Special Project of Wuhan (2020020601012256).

## Acknowledgments

We thank for the instrument help from Wuhan Regional Life Science Instrument Center.

## Conflict of interest

The authors declare that the research was conducted in the absence of any commercial or financial relationships that could be construed as a potential conflict of interest.

## Publisher’s note

All claims expressed in this article are solely those of the authors and do not necessarily represent those of their affiliated organizations, or those of the publisher, the editors and the reviewers. Any product that may be evaluated in this article, or claim that may be made by its manufacturer, is not guaranteed or endorsed by the publisher.

## Abbreviations

IRF, IFN regulatory factor; IRF-E, IRF-binding element; ISG, IFN-stimulated gene; ISRE, IFN-stimulated regulatory element, LGP2, laboratory genetics and physiology 2; MDA5, melanoma differentiation associated gene-5; poly(I:C), polyinosinic-polycytidylic acid; RIG-I, Retinoic acid inducible gene-I.
