# Supplementary material for: Function conservation and disparities of zebrafish and human LGP2 genes in fish and mammalian cells responsive to poly(I:C)
Source: Front Immunol. 2022 Aug 17;13:985792. doi: 10.3389/fimmu.2022.985792 (PMC9428467; doi:10.3389/fimmu.2022.985792)
Supplement: Supplementary file 1 [file DataSheet_1.docx]

**
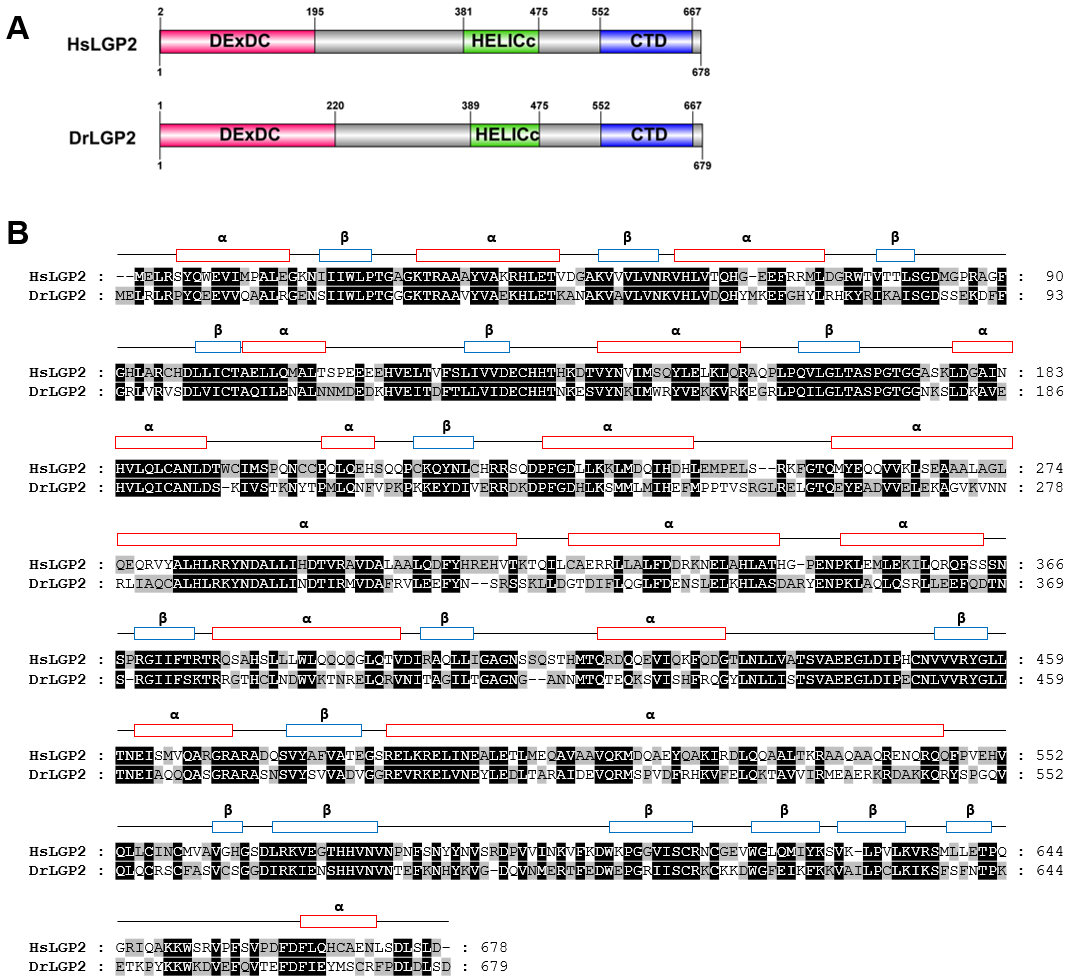
**

**Supplementary Figure 1.** **Domain schematic and sequence alignment of zebrafish and human LGP2s (related to Figure 1).**

1. Domain schematic of DrLGP2 and HsLGP2 were made with IBS software.
2. Comparison of amino acid sequences of zebrafish and human LGP2s was made by GENEDOC. The symbols including α-helices (red frame), β-strands (blue frame) indicates the secondary structures of zebrafish and human LGP2s, which were predicted by Alphfold. pdb in PyMol.


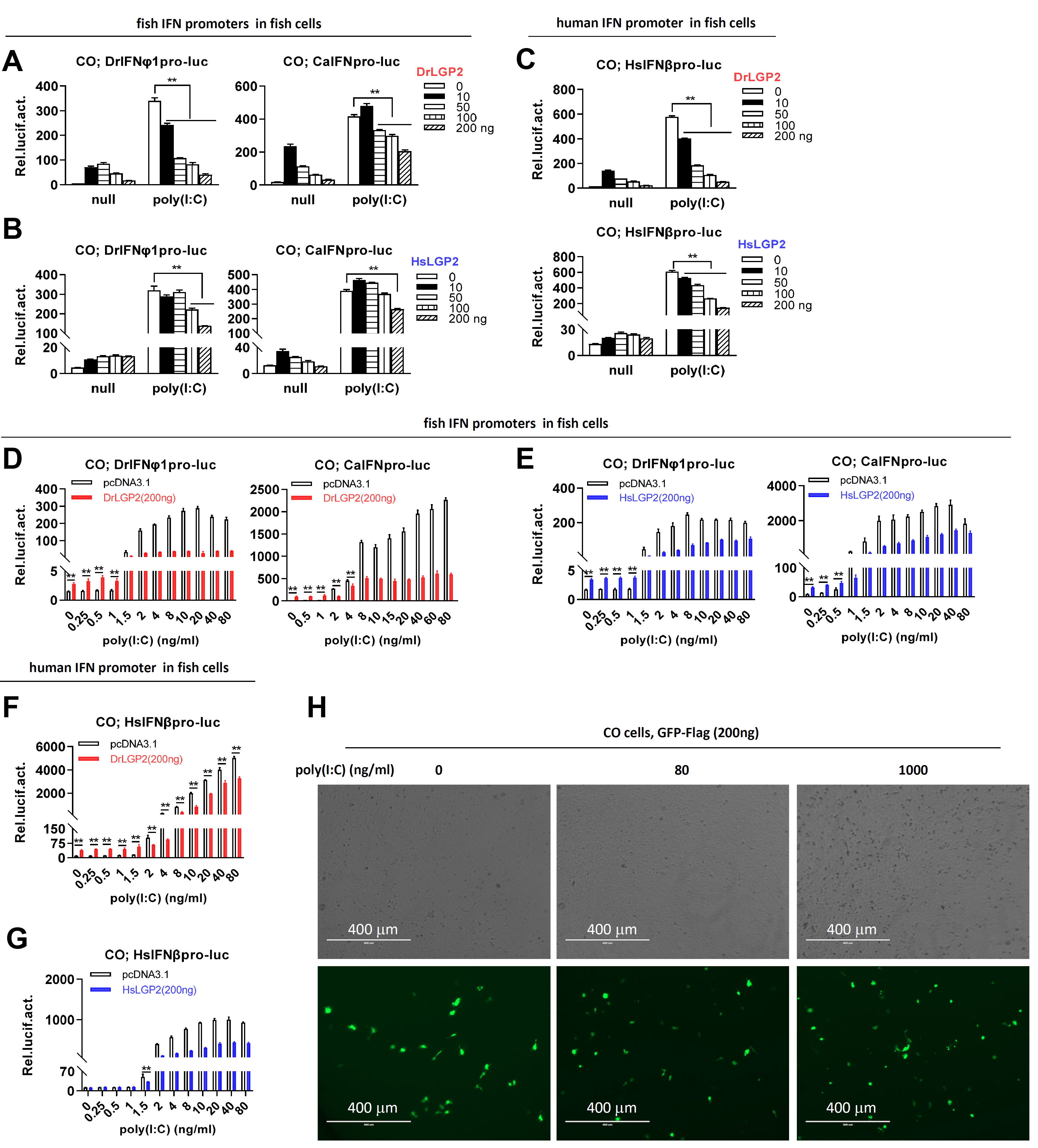


**Supplementary Figure 2. Zebrafish and human LGP2s play antithetic roles in regulating IFN response by poly(I:C) in fish cells (related to Figure 3).**

(A-C) DrLGP2 and HsLGP2 downregulated fish IFN and human IFN promoter activation by poly(I:C) at a high concentration of 1 μg/ml in CO cells. CO cells seeded in 24-wells plates were co-transfected with DrIFNφ1pro-luc or CaIFNpro-luc (A and B), or HsIFNβpro-luc (200 ng each) (C), together with DrLGP2 or HsLGP2 at increasing doses (0, 10, 50, 100, 200 ng). 24 h later, cells were transfected with 1 μg/ml poly(I:C) for another 24 h, followed by luciferase assays. *P* values were calculated using ANOVA. ***P*<0.01.

(D-G) DrLGP2 and HsLGP2 switched a first positive role to a following negative one in regulating IFN response by increasing concentrations of poly(I:C) in CO cells. CO cells seeded overnight in 24-wells plates were co-transfected with DrIFNφ1pro-luc or CaIFNpro-luc (D and E), or HsIFNβpro-luc (F and G), together with DrLGP2 (D and F) or HsLGP2 (E and G) (200 ng each). 24 h later, cells were transfected with poly(I:C) at increasing doses for another 24 h, followed by luciferase assays. *P* values were calculated using Student's t-test. ***P*<0.01.

(H) CO cells seeded in 24-wells plates were transfected with GFP-Flag (200 ng in 0.5 ml), and 24 h later, cells were transfected with increasing concentrations (0, 80, 1000 ng/ml) of poly(I:C) for another 24 h, followed by taking pictures with a confocal microscope [ZEN Blue Lite confocal system. Objectives: ×40; analysis software: ZEN 2.3 (blue edition)].


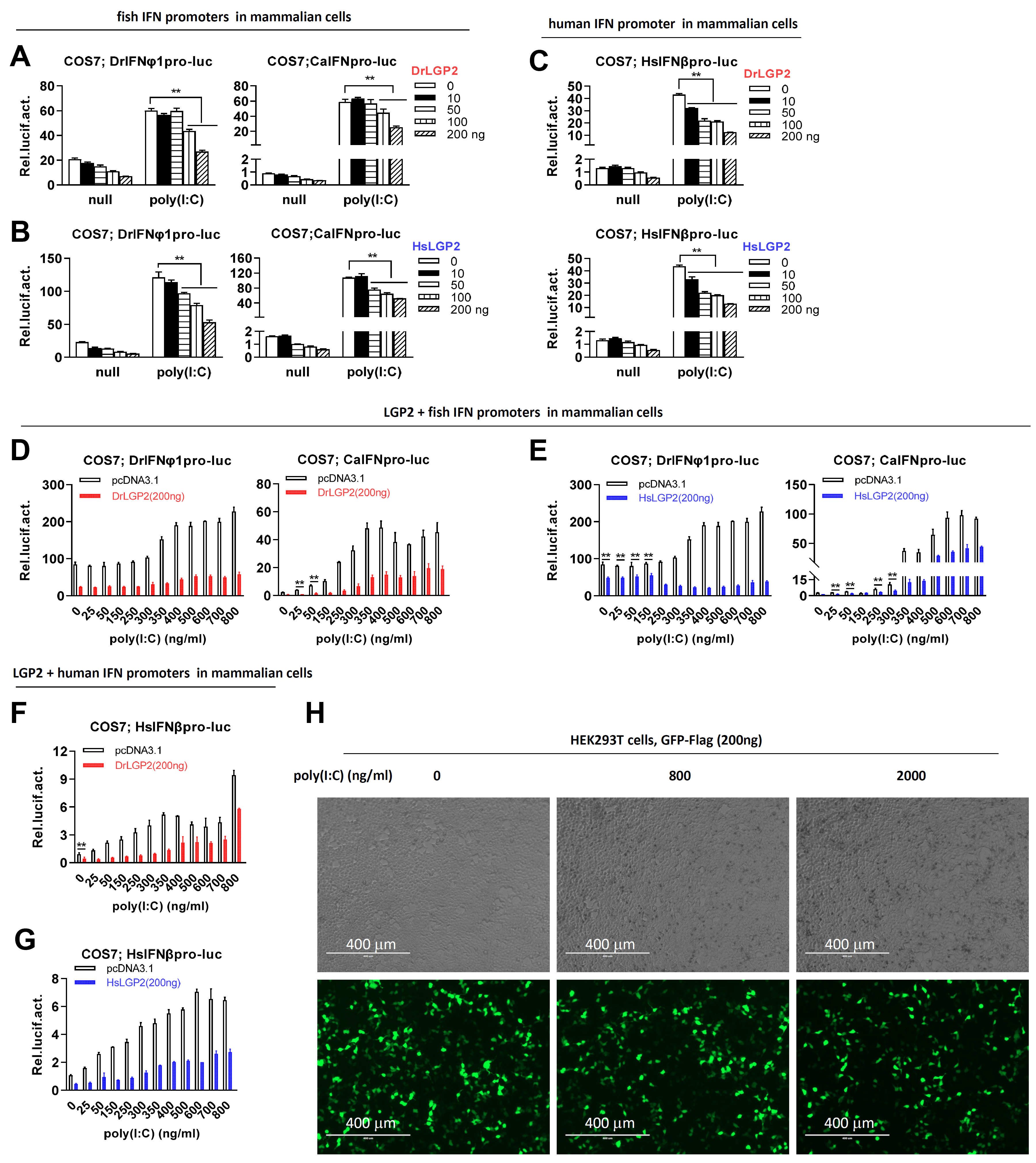


**Supplemental Figure 3. Zebrafish and human LGP2s play a negative role in regulating IFN response by high concentrations of poly(I:C) in mammalian cells (related to Figure 4).**

(A-C) DrLGP2 and HsLGP2 downregulated fish IFN and human IFNβ promoter activation by poly(I:C) at a high concentration of 2 μg/ml in COS7 cells. COS7 cells seeded in 24-wells plates were co-transfected with DrIFNφ1pro-luc or CaIFNpro-luc (A and B), or HsIFNβpro-luc (200 ng each) (C), together with DrLGP2 or HsLGP2 at increasing doses (0, 10, 50, 100, 200 ng). 24 h later, cells were transfected with 2 μg/ml poly(I:C) for another 24 h, followed by luciferase assays. *P* values were calculated using ANOVA. ***P*<0.01.

(D-G) Titration of poly(I:C) revealed a negative regulation of zebrafish and human LGP2s on IFN response in COS7 cells. COS7 cells seeded in 24-wells plates were co-transfected with HsIFNβpro-luc F and G), or with DrIFNφ1pro-luc or CaIFNpro-luc (D and E), together with DrLGP2 (D and F) or HsLGP2 (E and G) (200 ng each). Renilla vector (pRL-TK, 0.2 ng) was transfected as internal control. 24 h later, cells were transfected with poly(I:C) at increasing doses for another 24 h, followed by luciferase assays. *P* values were calculated using Student's t-test. ***P*<0.01.

(H) HEK293T cells seeded in 24-wells plates were transfected with GFP-Flag (200 ng in 0.5 ml), and 24 h later, cells were transfected with increasing concentrations (0, 800, 2000 ng/ml) of poly(I:C) for another 24 h, followed by taking pictures with a confocal microscope [ZEN Blue Lite confocal system. Objectives: ×40; analysis software: ZEN 2.3 (blue edition)].


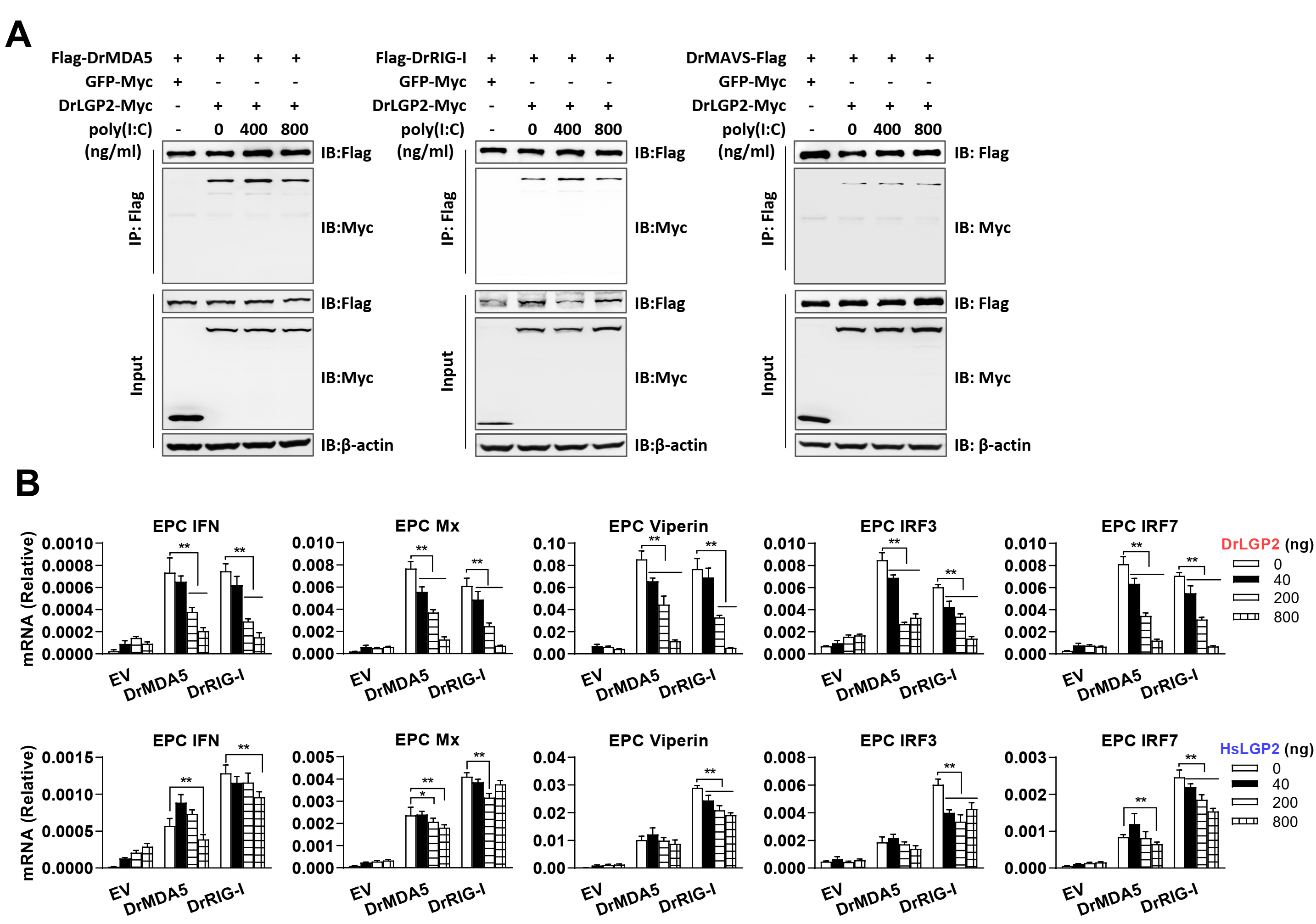


**Supplemental Figure 4. Zebrafish LGP2 binds to DrMDA5, DrRIG-I and DrMAVS, and induces transcript expression of cellular IFN gene and ISGs in EPC cells (related to Figure 6).**

1. Zebrafish LGP2 bound to DrMDA5, DrRIG-I and DrMAVS independently of poly(I:C) by Co-IP assays. HEK293T cells seeded in 10 cm dishes overnight were transfected with LGP2-myc, together with Flag-DrMDA5 (A), Flag-DrRIG-I (B), DrMAVS-Flag (C) (5 μg each), in the presence or absence of poly(I:C) at increasing doses (0, 400, 800 ng/ml) for 24 h. Cell lysates were immunoprecipitated with anti-Flag Ab, followed by western blot analysis of the immunoprecipitates with anti-myc Ab.
2. Overexpression of LGP2 induced the transcription of IFN and ISGs in EPC cells. EPC cells seeded in 6-wells plates were transfected with DrMAD5 or DrRIG-I (800 ng), DrLGP2 or HsLGP2 at increasing doses (0, 40, 200, 800 ng) for 24 h, followed by RT-PCR detection of *ifn* and *mx*, *viperin*, *irf3*, *irf7* mRNA. *P* values were calculated using ANOVA. ***P*<0.01, **P*<0.05.


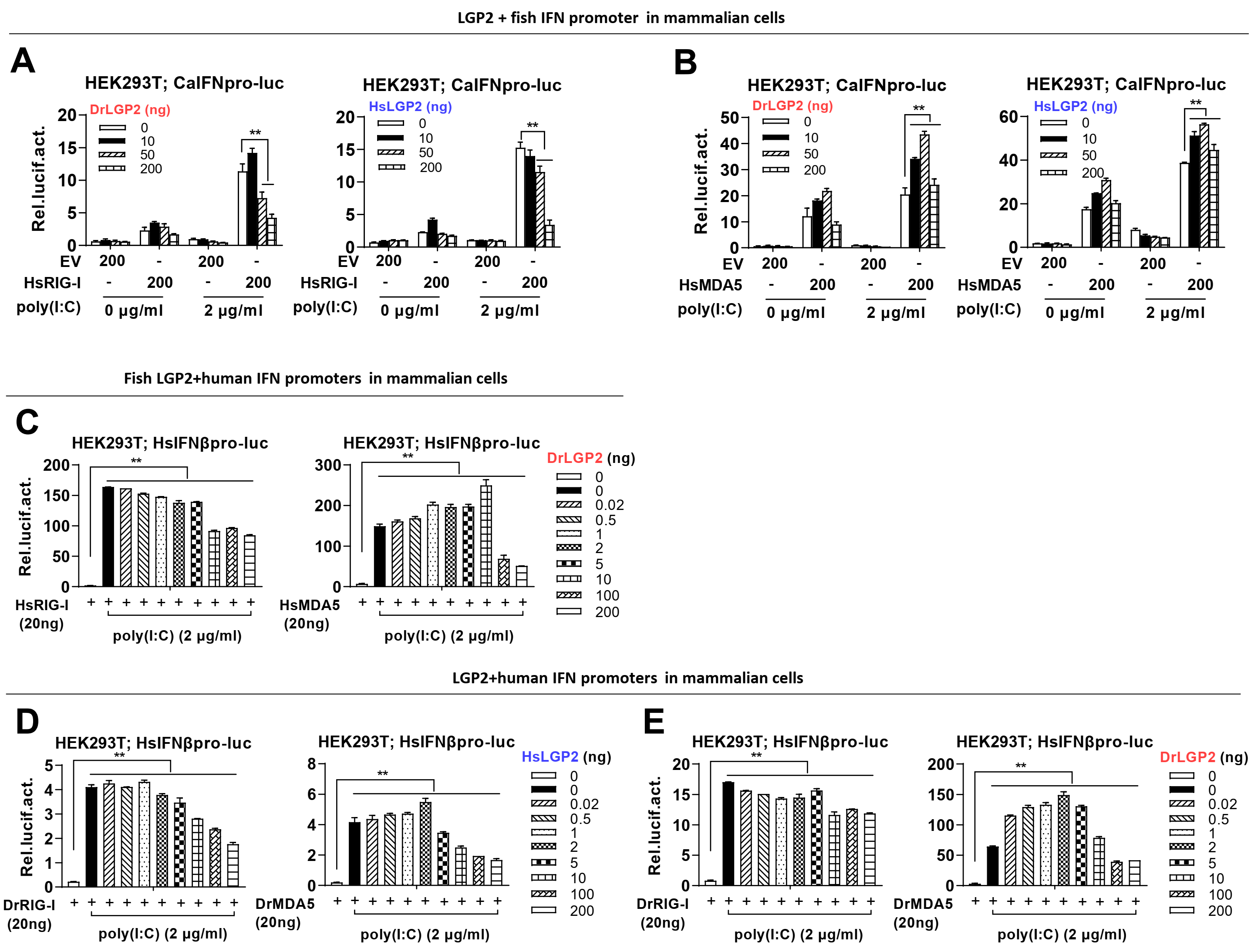


**Supplementary Figure 5.** **Zebrafish and human LGP2s promote MDA5 signaling and downregulate RIG-I signaling under different concentrations of poly(I:C) in mammalian cells (related to Figure 7).**

(A-B) DrLGP2 and HsLGP2 promoted fish IFN activation by HsMDA5 at a high dose and downregulated IFN response by HsRIG-I at a high dose in mammalian cells. HEK293T cells seeded in 24-wells plates were transfected with CaIFNpro-luc, DrLGP2 or HsLGP2 at increasing doses, together with HsRIG-I (A) or HsMAD5 (B) (200 ng each). 24 h later, cells were transfected again with or without poly(I:C) (2 μg/ml) for another 24 h, followed by luciferase assays.

(C) Titration of HsLGP2 expression revealed differential regulation of RIG-I- and MDA5-triggered IFN signaling by HsLGP2 in the presence of poly(I:C) in mammalian cells. HEK293T cells seeded in 24-wells plates were transfected with HsIFNβpro-luc, HsRIG-I or HsMAD5, together with the increasing doses of DrLGP2, 24 h later, cells were transfected again with or without poly(I:C) at 2 μg/ml for another 24 h, followed by luciferase assays.

(D-E) Titration of HsLGP2 expression revealed that LGP2-promoted DrMDA5 signaling and downregulated DrRIG-I signaling in the presence of poly(I:C) at 2 μg/ml in mammalian cells. HEK293T cells seeded in 24-wells plates were transfected with HsIFNβpro-luc, HsRIG-I or HsMAD5 (200 ng each), together with increasing doses of HsLGP2 (D) and DrLGP2 (E). 24 h later, cells were transfected with or without poly(I:C) at 2 μg/ml for another 24 h, followed by luciferase assays. *P* values were calculated using ANOVA. ***P*<0.01.

| Primer names | Sequences (5’ to 3’) | Applications |
| --- | --- | --- |
| HsLGP2-F (EcoRV) | GTGGAATTCTGCAGATGCCACCATGGAGCTTCGGTCCTACC | Plasmid construction |
| HsLGP2-R (EcoRV) | GCCACTGTGCTGGATGGTCCAGGGAGAGGTCC |  |
| HsIRF3-F (EcoRV) | GTGGAATTCTGCAGATGCCACCATGGGAACCCCAAAGCCACG |  |
| HsIRF3-R (EcoRV) | GCCACTGTGCTGGATGCTCTCCCCAGGGCCCTGGAAATC |  |
| HsMDA5-F (EcoRV) | GTGGAATTCTGCAGATGCCACCATGTCGAATGGG TATTCCACAGACG |  |
| HsMDA5-R (EcoRV) | GCCACTGTGCTGGATATCCTCATCACTAAATAAACAGC |  |
| HsRIG-I-F (EcoRV) | GTGGAATTCTGCAGATGCCACCATGACCACCGAGCAGCGAC |  |
| HsRIG-I-R (EcoRV) | GCCACTGTGCTGGATTTTGGACATTTCTGCTGGATC |  |
| EPC-Actin-F | CAGATCATGTTTGAGACC | RT-PCR  (EPC) |
| EPC-Actin-R | ATTGCCAATGGTGATGAC |  |
| EPC-IFN-F | ATGAAAACTCAAATGTGGACGTA |  |
| EPC-IFN-R | GTTTCCACCCATTTCCTTAAGGAG |  |
| EPC-Mx-F | GGCTGGAGCAGGTGTTGGTATC |  |
| EPC-Mx-R | CAGTTCCCTTATCCACCAGATCC |  |
| EPC-Viperin-F | AGCGAGGCTTACGACTTCTG |  |
| EPC-Viperin-R | CACCAGGAACAAACTTTATGCAG |  |
| EPC-IRF3-F | GGACGAGGAAAGCGTGTTCTC |  |
| EPC-IRF3-R | GTGAAATCTGCCCCAAACCACC |  |
| EPC-IRF7-F | AAAGTCTTCGTCAGCACCAGCG |  |
| EPC-IRF7-R | CTCTCCGAAGCACAGGTAGATGGT |  |
| DrLGP2-F | TGCAGTGCTCGTCAATAAGGTGC |  |
| DrLGP2-R | CATTCATCTATGACCAGTAGGG |  |
| HsGAPDH-F | TCAAGAAGGTGGTGAAGCAG | RT-PCR  (HEK293T) |
| HsGAPDH-R | GAGGGGAGATTCAGTGTGGT |  |
| HsISG15-F | TCCTGGTGAGGAATAACAAGGG |  |
| HsISG15-R | GTCAGCCAGAACAGGTCGTC |  |
| HsISG56-F | TCAGGTCAAGGATAGTCTGGAG |  |
| HsISG56-R | AGGTTGTGTATTCCCACACTGTA |  |
| HsLGP2-F | GTCTACAACGTCATCATGAGC |  |
| HsLGP2-R | CTGTTTGCAAGGCTGTTGGC |  |

**Supplementary Table 1 Primers used in this study**
